# Exploring the Dominant Role of Atomic‐ and Nano‐Ruthenium as Active Sites for Hydrogen Evolution Reaction in Both Acidic and Alkaline Media

**DOI:** 10.1002/advs.202004516

**Published:** 2021-06-04

**Authors:** Lijie Zhang, Haeseong Jang, Yan Wang, Zijian Li, Wei Zhang, Min Gyu Kim, Dongjiang Yang, Shangguo Liu, Xien Liu, Jaephil Cho

**Affiliations:** ^1^ State Key Laboratory Base of Eco‐Chemical Engineering College of Chemical Engineering Qingdao University of Science and Technology Qingdao 266042 P. R. China; ^2^ Department of Energy Engineering and School of Energy and Chemical Engineering Ulsan National Institute of Science and Technology (UNIST) Ulsan 44919 South Korea; ^3^ Electron Microscopy Center, and Key Laboratory of Automobile Materials MOE Jilin University Changchun 130012 China; ^4^ Beamline Research Division Pohang Accelerator Laboratory (PAL) Pohang 37673 Korea; ^5^ School of Environmental Science and Engineering State Key Laboratory of Bio‐fibers and Eco‐textiles Collaborative Innovation Center of Marine Biobased Fibers and Ecological textiles Institute of Marine Biobased Materials Qingdao University Shandong 266071 P. R. China

**Keywords:** electrocatalysts, hydrogen evolution reaction, metal–organic supramolecules, ruthenium, single atoms

## Abstract

Ru nanoparticles (NPs) and single atoms (SAs)‐based materials have been investigated as alternative electrocatalysts to Pt/C for hydrogen evolution reaction (HER). Exploring the dominant role of atomic‐ and nano‐ruthenium as active sites in acidic and alkaline media is very necessary for optimizing the performance. Herein, an electrocatalyst containing both Ru SAs and NPs anchored on defective carbon (Ru_SA+NP_/DC) has been synthesized via a Ru–alginate metal–organic supramolecules conversion method. Ru_SA+NP_/DC exhibits low overpotentials of 16.6 and 18.8 mV at 10 mA cm^−2^ in acidic and alkaline electrolytes, respectively. Notably, its mass activities are dramatically improved, which are about 1.1 and 2.4 times those of Pt/C at an overpotential of 50 mV in acidic and alkaline media, respectively. Theoretical calculations reveal that Ru SAs own the most appropriate H* adsorption strength and thus, plays a dominant role for HER in acid electrolyte, while Ru NPs facilitate the dissociation of H_2_O that is the rate‐determining step in alkaline electrolyte, leading to a remarkable HER activity.

## Graphical abstract

Defective carbon anchored Ru single atoms (SAs) and nanoparticles (NPs) is prepared, and exhibits super hydrogen evolution reaction (HER) activity in both acidic and alkaline media. Theoretical calculations reveal that Ru SAs are inclined to adsorption of H* that is critical for acidic HER, and Ru NPs facilitate the dissociation of H_2_O that is the rate‐determining step in alkaline medium.

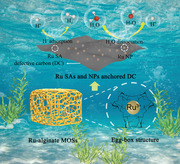

## 1 Introduction

The hydrogen evolution reaction (HER) is a fundamental electrochemical process occurring during water electrolysis, and the resulted hydrogen is most promising clean and renewable energy. However, its utilization is limited by its high overpotential and sluggish kinetics.^[^ ^1^, ^2^ ^]^ To date, precious metal Pt has been the most important component of HER electrocatalysts in both acidic and alkaline electrolytes. However, considering the high cost of Pt, exploring cost‐effective and highly active alternatives to the metal is urgent. Recently, Ru has drawn significant attention as a cost‐effective alternative to Pt, as the hydrogen bond strengths of both the metals are similar.^[^ ^3^, ^4^, ^5^ ^]^ Many highly efficient and stable Ru‐based electrocatalysts have been reported for the HER, such as Ru nanoparticles (NPs) in nitrogenated holey C_2_N layers (Ru@C_2_N) and graphene nanoplatelets (Ru@GnP).^[^ ^6^, ^7^ ^]^ To further enhance atom utilization efficiency, single atom catalysts (SACs) were studied for the HER in both acid and alkaline media.^[^ ^8^, ^9^ ^]^ For instance,

Ru single atoms (SAs) dispersed on amorphous phosphorus nitride imide nanotubes have been found to exhibit excellent HER activity and stability in acidic media.^[^ ^10^ ^]^ In acidic media, the HER mainly involves the adsorption of H* and generation and desorption of H_2_.^[^ ^11^ ^]^ The strength of H* adsorption determines the HER catalytic activity. However, the mechanism is significantly different in an alkaline medium. Before the adsorption of H*, an initial step of H_2_O dissociation (H_2_O + *e*− → H* + OH^−^) is required to break the H—OH bond, which would then generate H*.^[^ ^12^ ^]^ The high energy barrier for H_2_O dissociation retards the HER kinetics in alkaline media by two or three orders of magnitude compared to that in acidic media, and this determines the overall reaction rate. Although isolated atoms can adsorb H* moderately, it may be insufficient for H_2_O dissociation. This, in turn, hinders the generation of H*, thus lowering the alkaline HER kinetics.^[^ ^13^, ^14^ ^]^ Although some Ru SACs with good alkaline HER activities have been reported, these catalysts usually contain considerable amounts of NPs.^[^ ^15^, ^16^, ^17^ ^]^ Previous studies have shown that NPs may be more active for alkaline HER.^[^ ^7^, ^14^, ^18^, ^19^ ^]^ In this regard, an electrocatalyst comprising both SAs and NPs is expected to reduce the metal usage and at the same time, ensure super catalytic activities in both acidic and alkaline media. Recently, Kim and Tiwari et al. implanted Ru SAs and nitrided‐Ru NPs on N‐doped‐graphitic matrix, and superior HER performances compared to commercial Pt/C catalysts were observed in both acidic and alkaline media,^[^ ^20^ ^]^ and the adsorption of H* is emphasized for the reaction mechanism.

**Figure 1:**
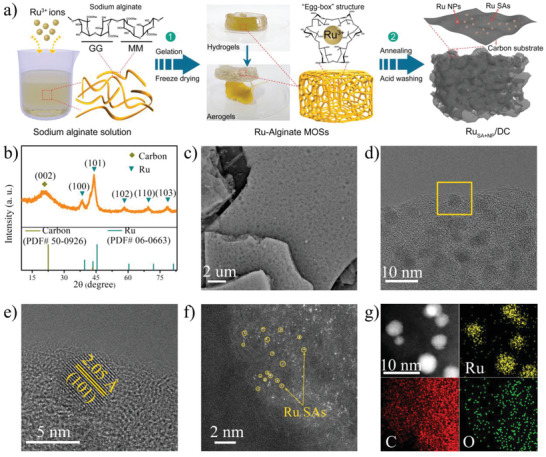
a) Schematic diagram of the synthetic route. b) XRD pattern, c) SEM image, d) TEM, e) HRTEM image, f) HAADF‐STEM image, and g) EDS mapping images of the synthesized Ru_SA+NP_/DC electrocatalyst.

Sodium alginate is a cheap ocean‐sourced polysaccharide extracted from abundant brown alga and is rich in hydrophilic groups such as —OH and —COO—.^[^ ^21^ ^]^ Sodium alginate can coordinate with multivalent metal ions (such as Co^2+^, Ni^2+^, and Ru^3+^) to form a unique “egg‐box” structured metal–organic supramolecule (MOSs), as described in **Figure** **1**a.^[^ ^22^, ^23^ ^]^ Such a three dimensional (3D) architecture allows an efficient dispersion and immobilization of the metal ions in the framework. Importantly, the “egg‐box” structure of MOSs are adjustable, such that metal ions can be partially exchanged with other cations (such as H^+^ and Zn^2+^),^[^ ^24^, ^25^ ^]^ thereby serving as fences to expand the spatial distance between the target metal ions. This, in turn, could effectively prevent metal agglomeration. As evident from our previous studies,^[^ ^26^, ^27^ ^]^ this feature allows the controllable synthesis of metal (SAs and NPs)/carbon nanocomposites through carbonization of MOSs by tuning the ratios of the metal ions in the “egg‐box.” Moreover, for an electrocatalyst including Ru SAs and NPs, it is very important to distinguish the role of Ru SAs and NPs for the HER in both acid and alkaline media.

In this study, we synthesized a Ru‐based electrocatalyst containing both SAs and NPs anchored on defective carbon (Ru_SA+NP_/DC) by simply carbonizing Ru–alginate MOSs. The synthesized Ru_SA+NP_/DC electrocatalyst exhibits ultralow *η*_10_ of 16.6 and 18.8 mV in acidic and alkaline media, respectively, comparable or even surpassing those of the commercial Pt/C electrocatalysts (16.5 and 32.2 mV, respectively) and most of the recently reported electrocatalysts. Notably, it exhibits remarkably high mass activities that are about 1.1 and 2.4 times that of 20 wt% Pt/C electrocatalysts at an overpotential of 50 mV in acidic and alkaline media, respectively. Theoretical calculations reveal that although isolated Ru atoms on defective carbon (DC) possess optimal H* binding strength, exhibiting remarkable acidic HER activity, they cannot efficiently dissociate H_2_O molecules, thus leading to sluggish kinetics in alkaline electrolytes. On the other hand, Ru NPs on DC can efficiently dissociate H_2_O to H*, thus increasing the alkaline HER activity remarkably.

## 2 Result and Discussion

Schematic diagram of the synthetic route is shown in Figure 1a. First, Ru–alginate MOSs hydrogels were obtained by a simple self‐assembly method using sodium alginate and ruthenium chloride as precursors, which further transformed into Ru–alginate MOS aerogels upon freeze drying.^[^ ^28^, ^29^ ^]^ The Ru_SA+NP_/DC composite was prepared by annealing Ru–alginate MOSs aerogels under Ar atmosphere at 1000 °C for 1.5 h. More details can be found in the Experimental Section of Supporting Information. X‐ray diffraction (XRD) pattern of Ru_SA+NP_/DC clearly confirms the conversion of Ru^3+^ ions to metallic Ru (Figure 1b and Figure S1, Supporting Information). The broad peak from 22° to 25° is attributed to the carbon generated from the carbonization of alginate macromolecules. The other diffraction peaks at 38.4°, 44.0°, 58.3°, 69.4°, and 78.4° correspond to the (100), (101), (102), (110), and (103) planes, respectively, of Ru NPs (JCPDS 06–663). Field emission‐scanning electron microscopy (FESEM) image shows that Ru_SA+NP_/DC has a 3D foam board‐like structure with abundant pores on both surface and the interior (Figure 1c). This is attributed to the release of CO_2_ upon the decomposition of alginate during annealing.^[^ ^30^ ^]^ The porous structure exposes more active sites and provides large contact areas with the electrolyte. Transmission electron microscopy (TEM) images show that Ru NPs with a diameter of 2–5 nm is almost homogeneously distributed on the carbon substrate (Figure 1d and Figure S2, Supporting Information), consistent with the EDS analysis (Figures S3 and S4, Supporting Information). The selected‐area electron diffraction pattern can be well‐indexed to the (100), (101), (101), (110), (102), (103), and (200) planes of hexagonal Ru (Figure S5a, Supporting Information), respectively. In addition, two set of lattice fringes with interplanar spacings of 2.05 and 2.34 Å are observed in the high‐resolution TEM image (Figure 1e and Figure S5b, Supporting Information). These can be ascribed to the (101) and (100) facets of hexagonal Ru, respectively, in agreement with the XRD analysis. To investigate the Ru SAs in Ru_SA+NP_/DC, high‐angle annular dark field scanning transmission electron microscopy (HAADF‐STEM) images were captured. The large number of bright spots circled in yellow denotes the Ru SAs, which are atomically dispersed in the carbon matrix (Figure 1f). The defect carbon was identified from the Raman spectrum. All these analyses confirm the successful synthesis of Ru_SA+NP_/DC. Furthermore, Ru_SA_/DC was also prepared using the same method by decreasing the concentration of Ru^3+^. As shown in Figure S6, Supporting Information, no signals corresponding to Ru NPs were detected in the XRD pattern and TEM images, and rich bright dots corresponding to Ru SAs were observed in the HAADF‐STEM image, indicating the formation of Ru_SA_/DC. EDS mappings of Ru_SA+NP_/DC and Ru_SA_/DC (Figure 1g and Figure S6f, Supporting Information) show that all the elements, including Ru, C, and O, are uniformly distributed over the entire sample, wherein O is derived from the carbon substrate carbonized from the alginate.

**Figure 2:**
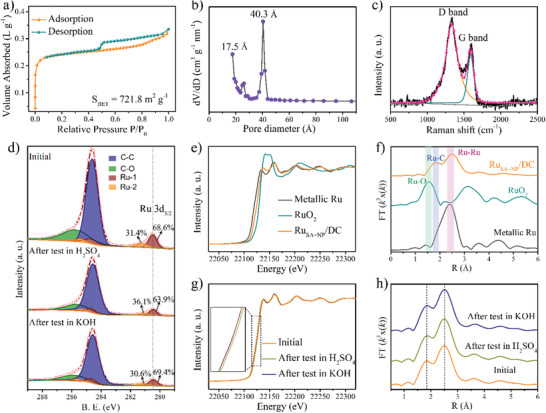
a) N_2_ adsorption–desorption isotherms, b) corresponding pore diameter distribution, and c) Raman spectrum of Ru_SA+NP_/DC. d) High‐resolution XPS spectra of Ru 3d and C 1s for samples before and after the HER. e) Ru K‐edge XANES and f) FT‐EXAFS spectra of Ru_SA+NP_/DC, metallic Ru, and RuO_2_. g) Ru K‐edge XANES spectra and h) FT‐EXAFS spectra of samples before and after the HER.

The surface area and pore size distributions of the samples were determined from the N_2_ adsorption–desorption isotherms (**Figure** **2**a and Figure S7, Supporting Information). The combination of type I and type IV adsorption isotherms and H4 hysteresis loop, suggests that the Ru_SA+NP_/DC catalyst has a highly porous structure with abundant macro‐, meso‐, and micro‐pores,^[^ ^31^ ^]^ in agreement with the FESEM images. The Brunauer–Emmett–Teller surface areas of all the Ru_SA+NP_/DC samples were in the range of 624.6–721.8 m^2^ g^−1^, and the corresponding Barret–Joyner–Halenda pore diameters were ∼17.5 and 40.3 Å, which is consistent with the adsorption–desorption isotherms. The high surface area and multimodal porous structure of Ru_SA+NP_/DC expose more number of active sites and facilitate the mass transport, which are beneficial for the catalytic process.^[^ ^32^ ^]^ The Raman spectra display two distinct peaks at about 1320 and 1600 cm^−1^ (Figure 2c and Figure S8, Supporting Information), corresponding to the characteristic D (defects or disordered carbon) and G bands (graphitic or ordered carbon), respectively, of the carbon matrix.^[^ ^33^, ^34^ ^]^ The high *I*_D_/*I*_G_ ratio suggests that abundant intrinsic defects are generated on the carbon framework carbonized of MOSs, providing rich trapping sites for Ru SAs via strong interaction between the *d* band state of the metal atoms and 2*π* antibond state of the carbon atoms.^[^ ^35^ ^]^

The structure and surface chemistry of the catalysts before and after the HER were first investigated by XRD and X‐ray photoelectron spectroscopy (XPS). The diffraction peaks corresponding to hexagonal Ru are clearly observed in the XRD patterns of the samples after the HER (Figure S9, Supporting Information), implying a perfect preservation of the Ru NPs and hence, an excellent structural stability of the catalyst. The XPS survey spectrum (Figure S10, Supporting Information) reveals the presence of Ru, C, and O in the samples. High‐resolution XPS spectra of Ru 3d and C 1s (Figure 2d) indicate a C—C peak at 284.6 eV and C—O peak at 285.8 eV in the C 1s spectrum;^[^ ^36^ ^]^ additionally, two kinds of Ru species are resolved. The first peak of Ru 3d_5/2_ is located at ∼280.4 eV, corresponding to metallic Ru^(0)^ (wine, Ru‐1). The second peak (orange, Ru‐2) is located at a relatively high energy, ∼281.1 eV, indicating a slightly positive‐charged Ru species, similar to Ru SAs observed previously.^[^ ^10^, ^37^ ^]^ This is consistent with the existence of both Ru NPs and Ru SAs in the catalyst, as evident from the TEM and HADDF‐STEM images. The NPs and SAs account for 68.6% and 31.4% of the total Ru content, respectively. After the catalysis, although the peak positions in the XPS spectra do not change, the peak proportions are different from those before the catalysis. After the HER in 0.5 m H_2_SO_4_, the relative content of Ru SA increased to 36.1%, whereas the relative content of metallic Ru^(0)^ decreased to 63.9%. In comparison, after the HER in 1 m KOH, the proportion of metallic Ru^(0)^ increased (69.4%), whereas that of Ru SAs decreased (30.6%). The metal NPs/SAs‐based electrocatalysts are generally suffered from the leaching or aggregation of active metal centers during the catalytic process. Due to the corrosiveness of acidic electrolyte, the Ru NPs are more likely to be leached during the catalytic process in 0.5 m H_2_SO_4_, diminishing the content of Ru NPs, thus leading to the increase of relative content of Ru SAs. Compared with 0.5 m H_2_SO_4_, the leaching of Ru NPs is alleviated in 1 m KOH, where the aggregation becomes dominant, giving rise to the increase of relative content of metallic Ru. These results are also demonstrated by content changes for Ru NPs and SAs before and after electrochemical test in 0.5 m H_2_SO_4_ and 1 m KOH (Table S1, Supporting Information).

To further investigate the chemical environment and electronic structures of the Ru species, X‐ray absorption near‐edge structure (XANES) and extended X‐ray absorption fine structure (EXAFS) spectroscopies were performed. Figure 2e shows the Ru K‐edge XANES spectra of Ru_SA+NP_/DC, Ru foil, and RuO_2_. The spectrum of Ru_SA+NP_/DC is very similar to that of the Ru foil but remarkably different from that of RuO_2_, demonstrating that metallic Ru is the dominant Ru species in Ru_SA+NP_/DC. In addition, the peak intensity of the white line of Ru_SA+NP_/DC is slightly higher than that of the Ru foil, indicating the slightly oxidized electronic structure of the Ru species due to the presence of Ru SAs. The Fourier transform (FT) of the EXAFS curves of Ru_SA+NP_/DC, Ru foil, and RuO_2_ are depicted in Figure 2f. The obvious main peak centered at 2.4–2.6 Å in the spectrum of Ru_SA+NP_/DC can be ascribed to the Ru–Ru interactions in the Ru NPs, which are similar to that in the Ru foil. Besides, a minor peak at 1.8–1.9 Å is also observed in the spectrum of Ru_SA+NP_/DC, and this can be attributed to the backscattering between Ru SAs and light elements, such as the C and O elements in the material. As reflected by the spectrum of RuO_2_, the Ru—O path peak is located at about 1.55 Å, which is quite different from that of Ru_SA+NP_/DC (1.8–1.9 Å), indicating that the minor peak is caused not by the backscattering between Ru and O, but by the backscattering between Ru and adjacent C. This is also in agreement with a previous report on atomically dispersed materials,^[^ ^17^ ^]^ thereby demonstrating the formation of Ru SAs. According to the fitting results and detailed parameters (Figure S11 and Tables S2, Supporting Information), the coordination numbers of Ru—C are in the range of 3–4 for both samples before and after HER test, indicating that the structures of Ru SAs are RuC_3_ and RuC_4_ moieties. Therefore, Ru NPs and SAs coexist in the catalyst, which is consistent with the XRD, TEM, HAADF‐STEM, and XPS analyses. After the HER in 0.5 m H_2_SO_4_, the XANES curve was shifted toward high photon energy, indicating an increase in the content of Ru SAs with high valence state. On the other hand, in 1 m KOH, a small shift toward low photon energy was observed due to the increased Ru NPs content (Figure 2g). These results are in accordance with the above XPS analysis. The FT‐EXAFS spectra (Figure 2h) of all the samples before and after the HER had the same shape, without any change in the peak positions. This demonstrates their similar local atomic arrangements and excellent catalytic stability.^[^ ^38^ ^]^

**Figure 3:**
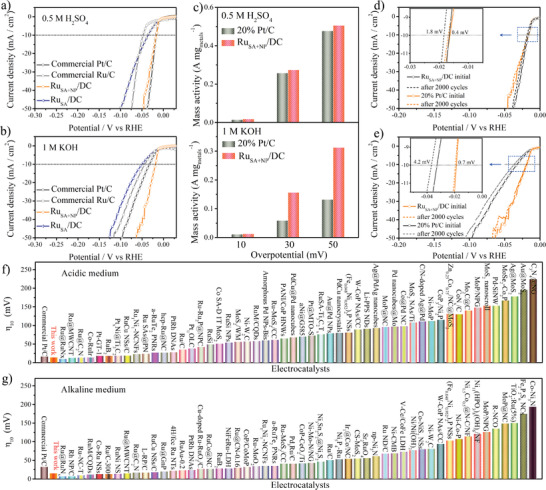
HER polarization curves of Ru_SA+NP_/DC, Ru_SA_/DC, commercial Pt/C, and commercial Ru/C in a) 0.5 m H_2_SO_4_ and b) 1 m KOH. c) Comparison of mass activities of Ru_SA+NP_/DC and commercial Pt/C at overpotentials of 10, 30, and 50 mV in 0.5 m H_2_SO_4_ and 1 m KOH. Stability test of Ru_SA+NP_/DC and commercial Pt/C before and after 2000 cycles in d) 0.5 m H_2_SO_4_ and e) 1 m KOH. Comparison of *η*_10_ values for Ru_SA+NP_/DC, 20% commercial Pt/C and other recently reported HER electrocatalysts in f) acidic and g) alkaline media.

The HER catalytic performances of the samples were investigated in both 0.5 m H_2_SO_4_ and 1 m KOH. Apparently, the alginate carbonized DC displays a poor HER activity (Figure S12a, Supporting Information) in 0.5 m H_2_SO_4_ (*η*_10_ > 500 mV). When isolated Ru atoms were anchored on DC, the catalytic activity increased dramatically with increasing content of Ru atoms (Figure S12a, Supporting Information). Theoretically, the activity can be further improved by increasing the Ru content in Ru_SA_/DC.^[^ ^26^, ^39^ ^]^ However, due to the large cohesive energy, Ru SAs easily aggregate into clusters/NPs, resulting in a low metal loading in Ru_SA_/DC. Since we also need to prevent the aggregation, which is a huge challenge in the synthesis of SACs, the Ru content could not be increased further.^[^ ^40^, ^41^ ^]^ The lowest *η*_10_ of Ru_SA_/DC attained was 35.5 mV when the Ru content was 0.80 wt%, as measured by inductively coupled plasma (ICP). Notably, Ru_SA_/DC exhibited superior HER activity compared to commercial Ru/C catalysts (46.9 mV), demonstrating the significant advantages of SAs in catalyzing the acidic HER. Besides Ru SAs, a further increase in the Ru content leads to the formation of Ru NPs, forming Ru_SA+NP_/DC. The HER activity of Ru_SA+NP_/DC is remarkably enhanced due to the considerable increase in the numbers of active sites. The best Ru_SA+NP_/DC sample displays an ultralow *η*_10_ of 16.6 mV in 0.5 m H_2_SO_4_ (**Figure** **3**a and Figures S13–S15, Supporting Information), which is comparable to that of the commercial Pt/C catalyst (16.5 mV). The Ru content in this sample of Ru_SA+NP_/DC was measured to be 11.8 wt% by ICP. Surprisingly, the acidic HER activity of Ru_SA+NP_/DC outperforms most of the recently reported HER electrocatalysts (Figure 3f and Table S3, Supporting Information).

Similar to that in acidic media, the HER activity of Ru_SA_/DC was superior to that of DC in 1 m KOH, and it improved with increasing Ru content due to the increased number of active sites (Figure S12b, Supporting Information). The best Ru_SA_/DC sample exhibits a low *η*_10_ of 51.4 mV. Unlike that in an acidic medium, the HER activity of Ru_SA_/DC in 1 m KOH is inferior to that of the commercial Ru/C catalysts (43.9 mV), indicating that Ru SAs may be insufficient for the HER in alkaline media. With the formation of Ru NPs in the catalyst, the HER activity in the alkaline media was dramatically improved. Thus, Ru NPs may have a higher contribution compared to Ru SAs toward the HER activity in alkaline media. Figure 3b and Figures S16 and S17, Supporting Information, suggest that the best *η*_10_ of Ru_SA+NP_/DC is as low as 18.8 mV, which significantly outperforms that of the commercial Pt/C catalyst (32.2 mV). It is worth mentioning that this value is far beyond those of most of the other Pt or non‐Pt electrocatalysts recently reported (Figure 3g and Table S4, Supporting Information). Therefore, Ru_SA+NP_/DC is an outstanding HER electrocatalyst in both acidic and alkaline media.

The HER kinetics were further investigated from the Tafel plots. Figures S13–S14b and S16–S17b, Supporting Information, depict that the smallest Tafel slopes, 28.7 and 35.8 mV dec^−1^, were obtained for Ru_SA+NP_/DC in the acidic and alkaline media, respectively. These are lower than those of the commercial Pt/C catalyst (29.5 and 48.2 mV dec^−1^), indicating the fastest reaction kinetics of Ru_SA+NP_/DC in the acidic and alkaline media.^[^ ^42^ ^]^ The Tafel slopes suggest that Ru_SA+NP_/DC follows the Volmer–Tafel mechanism in acidic media, with the Tafel reaction, H* + H* → H_2_, determining the overall reaction, whereas it follows the Volmer–Heyrovsky mechanism in alkaline media, with the Heyrovsky reaction, H_2_O + *e* ^−^ + H* → H_2_ + OH^−^, being the rate‐determining step.^[^ ^11^, ^12^ ^]^ To investigate the reaction mechanism, it is necessary to account for the effect of acidic and alkaline media. To quantify the intrinsic active surface area of the electrocatalysts, the electrochemically active surface area (ECSA) was measured by double layer capacitance (*C*_dl_) using a cyclic voltammetry method.^[^ ^43^ ^]^ Obviously, the highest *C*_dl_ values of 77.6 and 121.6 mF cm^−2^ were observed for Ru_SA+NP_/DC in the acidic and alkaline media (Figures S18–S22, Supporting Information), respectively. These correspond to ECSAs of 2217.1 and 3040.0 cm^2^ in the acidic and alkaline media, which are about 8.7 and 3.3 times higher, respectively, than those of the commercial Pt/C catalyst. Moreover, the underpotential deposition method was used to quantitatively analyze the number of active sites of Ru_SA+NP_/DC and commercial Pt/C (Figure S23, Supporting Information). After calculation, the active site density of Ru_SA+NP_/DC is 1.13 × 10^14^ sites per cm^2^, whereas it is 9.94 × 10^13^ sites per cm^2^ for that of Pt/C, demonstrating that the Ru_SA+NP_/DC possesses more active sites than Pt/C.

Mass activity is an important factor in determining the catalyst cost. Apparently, Ru_SA+NP_/DC exhibits higher mass activities than Pt/C in both acidic and alkaline media (Figure S24, Supporting Information). Specifically, at overpotentials of 10, 30, and 50 mV, the mass activities of Ru_SA+NP_/DC are 0.03, 0.28, and 0.53 A mg_Ru_^−1^ in acidic media and 0.02, 0.16, and 0.31 A mg_Ru_^−1^ in alkaline media (Figure 3c), respectively, which exceed those of Pt/C (0.02, 0.26, and 0.48 A mg_Pt_^−1^ [acidic media] and 0.01, 0.06, and 0.13 A mg_Pt_^−1^ [alkaline media], respectively). To assess the catalytic efficiency, the turnover frequency (TOF) of the catalyst was calculated by assuming that all the metal atoms in the catalyst are active sites participating in the HER.^[^ ^35^ ^]^ The least TOF for Ru_SA+NP_/DC at overpotentials of 10, 20, and 30 mV were estimated to be 0.02, 0.14, and 0.27 H_2_ s^−1^ in 0.5 m H_2_SO_4_ and 0.01, 0.10, and 0.17 H_2_ s^−1^ in 1 m KOH (Figure S25, Supporting Information), respectively. These TOFs surpass those of Pt/C (0.015, 0.11, and 0.25 H_2_ s^−1^ in 0.5 m H_2_SO_4_ and 0.01, 0.03, and 0.06 H_2_ s^−1^ in 1 m KOH, respectively). Thus, besides the high geometric activity, Ru_SA+NP_/DC also shows desirable mass activity and catalytic efficiency, enhancing the material utilization efficiency during industrial upscaling.

To probe the catalytic stability, accelerated degradation tests were performed. Figure 3d shows that the polarization curve of Pt/C is negatively shifted after 2000 cycles in 0.5 m H_2_SO_4_, with 1.8 mV change in *η*_10_. In contrast, Ru_SA+NP_/DC exhibits a small negative shift of 0.4 mV. In 1 m KOH, a small negative shift of 0.7 mV in *η*_10_ is observed for Ru_SA+NP_/DC after 2000 cycles (Figure 3e), which is much smaller than that of the commercial Pt/C catalyst (4.2 mV). These results provide strong evidences of the remarkable stabilities of the Ru_SA+NP_/DC catalyst in acidic and alkaline media, and this is also confirmed by the corresponding *i*‐t tests (Figure S26, Supporting Information). Therefore, considering the high mass activity, high stability, and low price of Ru, it can be safely concluded that Ru_SA+NP_/DC has significant advantages over Pt/C in cost and catalytic performance.

To verify the actual application, a two‐electrode electrolyzer using Ru_SA+NP_/DC and commercial RuO_2_ as the cathode and anode, respectively (Ru_SA+NP_/DC‖RuO_2_), were assembled and tested in 1 m KOH. Figure S27a, Supporting Information, shows that Ru_SA+NP_/DC‖RuO_2_ requires a low cell voltage of 1.86 V to achieve a high current density of 100 mA cm^−2^, which is much smaller than that required by Pt/C‖RuO_2_ (1.96 V). Remarkably, it delivers a current density of 200 mA cm^−2^ at a cell voltage of 2.15 V, satisfying the requirements of industrial water splitting.^[^ ^44^ ^]^ Besides, Ru_SA+NP_/DC‖RuO_2_ exhibits excellent catalytic stability for water splitting. Figure S27b, Supporting Information, shows that after continuously operating for 30 h, Ru_SA+NP_/DC‖RuO_2_ retains 76.5% of the initial current even at a high current density of 100 mA cm^−2^, outperforming Pt/C‖RuO_2_ (71.2%). Thus, owing to the high activity and stability, Ru_SA+NP_/DC is a promising alternative HER electrocatalyst to Pt/C catalysts for practical water electrolysis.

**Figure 4:**
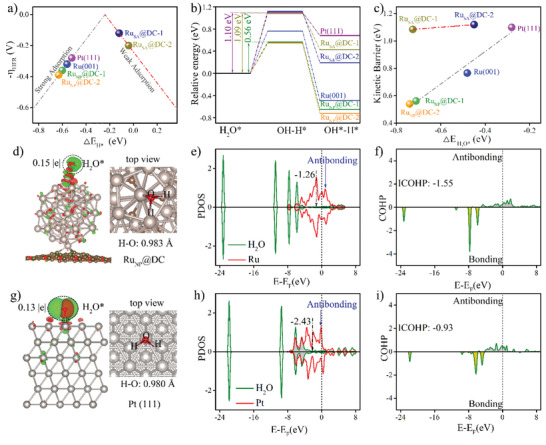
a) Volcano plot of theoretical *η*_HER_ versus Δ*E*_H*_. b) Kinetic barrier of H_2_O dissociation for different models. c) Correlation between Δ*E*_H2O*_ and energy barrier of H_2_O dissociation. d) Differential charge density distributions, e) PDOS, and f) COHP of active Ru atom for Ru_NP_@DC‐1 and adsorbed H_2_O. g) Differential charge density distributions, h) PDOS, and i) COHP of active Pt atom for Pt (111) and adsorbed H_2_O. For differential charge density distributions, the green and red regions represent positive and negative charges, respectively.

To gain further insights into the role of SA and NP in the HER activities in the acidic and alkaline media, density functional theory (DFT) calculations were performed. Based on the experimental analysis, two common types of defective graphene—single and double defect graphene (DC‐1 and DC‐2)—were employed as carbon substrates. Ru SA and Ru_55_ NP were separately anchored on DC‐1 and DC‐2, forming Ru_SA_@DC‐1 (RuC_3_), Ru_SA_@DC‐2 (RuC_4_), Ru_NP_@DC‐1, and Ru_NP_@DC‐2 models (Figure S28, Supporting Information). The Sabatier principle states that a good acidic HER electrocatalyst should bind the adsorbed H* moderately.^[^ ^45^, ^46^ ^]^ Thus, binding strength of the adsorbed H* species (Δ*E*_H*_) is first calculated. The volcano plot (**Figure** **4**a) shows that Ru_NP_@DC‐1, Ru_NP_@DC‐2, and Ru (001) exhibit more negative Δ*E*_H*_ values compared to Pt (111), indicating that H* binds more strongly to the surface of the Ru NPs than to the surface of Pt. This will hinder H_2_ desorption on the surface of the Ru NPs, leading to a relatively high overpotential for the HER (*η*_HER_). In comparison, Δ*E*_H*_ is largely weakened on Ru_SA_@DC‐1 and Ru_SA_@DC‐2, alleviating the difficulties in desorption and thereby lowering *η*_HER_ and taking them closer to the center of the volcano plot. Moreover, the Gibbs free energy of H* (Δ*G*_H*_) was calculated. A moderate Δ*G*_H*_ close to 0 eV is desired for a super acidic HER electrocatalyst.^[^ ^17^ ^]^ Figure S29, Supporting Information, shows that Ru_SA_@DC‐1 exhibits the most desirable Δ*G*_H*_ of 0.12 eV, which is slightly better than that of Ru_SA_@DC‐2 (0.20 eV), and significantly more promising than that of Ru_NP_@DC‐1 (−0.36 eV), Ru_NP_@DC‐2 (−0.39 eV), and Pt (111) (−0.28 eV), consistent with the volcano plot. Therefore, theoretically, Ru SA can catalyze HER in acidic media more efficiently. For an alkaline medium, despite the moderate Δ*G*_H*_, a low H_2_O dissociation barrier is essential for a superior alkaline HER electrocatalyst. Figure 4b shows that the energy barrier for H_2_O dissociation of Ru_SA_@DC‐1 and Ru_SA_@DC‐2 are as high as 1.09 and 1.12 eV, respectively, suggesting that Ru SA is insufficient for dissociating the H—OH bond, which hinders the H* formation and leads to sluggish alkaline HER kinetics. In contrast, Ru (001), Ru_NP_@DC‐1, and Ru_NP_@DC‐2 prominently reduce the energy barrier to 0.77, 0.56, and 0.54 eV, respectively, indicating that Ru NP can efficiently dissociate H_2_O, thereby facilitating the alkaline HER. Therefore, compared to Ru SA, Ru NP contributes more to the HER activity in an alkaline electrolyte. For the NP‐based models, a stronger adsorption free energy of H_2_O (Δ*E*_H2O*_) leads to lower energy barrier of H_2_O dissociation, according to the linear Brønsted–Evans–Polanyi relationship (Figure 4c).^[^ ^47^ ^]^ However, this rule is not applicable to the SA‐based models (Ru_SA_@DC‐1 and Ru_SA_@DC‐2) (Figures S30 and S31, Supporting Information) due to the steric hindrance effects,^[^ ^48^, ^49^ ^]^ demonstrating that the size and structure of the metal‐centered sites significantly affect the H_2_O adsorption and dissociation, resulting in different HER activities in the alkaline media. Consequently, the dominant roles of Ru SA and NPs in various media have been revealed from theoretical calculation combining experiments, particularly the water dissociation step has been taken into account, which distinguished from the previous literatures (Table S5, Supporting Information). Few‐atom clusters are bridge between SA and NP. Herein, to illustrate the contributions of Ru clusters, DFT calculations were performed on the model of four Ru atoms anchored defective carbon (Ru_cluster_@DC‐1). As shown in Figure S32, Supporting Information, both the Δ*G*_H*_ value and H_2_O dissociation barrier of Ru_cluster_@DC‐1 is between Ru_SA_@DC‐1 and Ru_NP_@DC‐1, suggesting that the HER activity of Ru cluster is between Ru SA and Ru NP in both acidic and alkaline media.

To gain insights into the effect of inherent electronic properties of Pt and Ru on H_2_O dissociation, differential charge density distribution analysis, Bader charge analysis, and projected density of states (PDOS) were performed on H_2_O‐adsorbed Ru_NP_@DC‐1 and H_2_O‐adsorbed Pt (111). From Figure 4d,g, it is obvious that for both Ru_NP_@DC‐1 and Pt (111), charge is transferred from metal atoms to H_2_O molecules. Due to this charge transfer, the H—O bond of the adsorbed H_2_O is significantly elongated. Compared with Pt (111), more charge transfer occurs on Ru_SA_@DC‐1 (0.15 |e|), and the adsorbed H_2_O molecule is more likely to dissociate owing to its longer H—O distance (0.983 Å). The PDOS (Figure 4e,h) depicts that the Pt 5d band center is located far from the Fermi level (−2.43), while the Ru 4d‐band center moves upward toward the Fermi level (−1.26). According to the *d*‐band center theory, this change will lower the occupancy of the antibonding state of the H_2_O‐adsorbed Ru_NP_@DC‐1 during the hybridization of the O 2p orbital with Ru 4d orbital (as indicated by the blue arrow), thereby increasing Δ*E*_H2O*_. The integrated‐crystal orbital Hamilton population value of Ru—O for Ru_NP_@DC‐1 is −1.55 (Figure 4f,i), which is much lower than that of Pt—O in Pt (111) (−0.93), further demonstrating the strong bonding between the active surface Ru and ligand O atoms. Consequently, compared with Pt, H_2_O is more easily adsorbed and dissociated to H* on the surface of the Ru NPs.

## 3 Conclusion

In conclusion, a Ru‐based electrocatalyst containing both Ru SAs and NPs anchored on DC was developed by simply annealing Ru–alginate MOSs. The Ru_SA+NP_/DC catalyst exhibits superior HER activities in acidic and alkaline media, with ultralow *η*_10_ of 16.6 and 18.8 mV, respectively. Notably, the mass activities of Ru_SA+NP_/DC at the overpotential of 50 mV are ∼1.8 and 4.4 times those of Pt/C in acidic and alkaline media, respectively. DFT calculations reveal the different roles of Ru SA and NPs in various media. Ru SAs efficiently optimize Δ*G*_H*_, leading to an outstanding HER activity in acidic media, while Ru NPs largely lower the energy barrier for H_2_O dissociation, facilitating the HER in alkaline media. Although the number of active sites and the activity of individual sites between Ru SA and NPs are difficult to be distinguished, we can get an enlightenment from this work; the amount of Ru NPs and SAs can be tuned in various media so that we can obtained the best optimized HER catalysts that is our purpose in future.

## Conflict of Interest

The authors declare no conflict of interest.

## Supporting information

Supporting Information

## Data Availability

Research data are not shared.
