# Supplementary material for: Exploring the Dominant Role of Atomic‐ and Nano‐Ruthenium as Active Sites for Hydrogen Evolution Reaction in Both Acidic and Alkaline Media
Source: Adv Sci (Weinh). 2021 Jun 4;8(15):2004516. doi: 10.1002/advs.202004516 (PMC8336516; doi:10.1002/advs.202004516)
Supplement: Supplementary file 1 — Supporting Information [file ADVS-8-2004516-s001.pdf]

© 2021 Wiley-VCH GmbH

ADVANCED SCIENCE
Open Access

Supporting Information

for *Adv. Sci.*, DOI: 10.1002/advs.202004516

Exploring the Dominant Role of Atomic- and Nano-Ruthenium as Active Sites for Hydrogen Evolution Reaction in Both Acidic and Alkaline Media

Lijie Zhang, Haeseong Jang, Yan Wang, Zijian Li, Wei Zhang, Min Gyu Kim, Dongjiang Yang, Shangguo Liu, Xien Liu,* and Jaephil Cho**

Supporting information

Exploring the Dominant Role of Atomic- and Nano-Ruthenium as Active Sites for Hydrogen Evolution Reaction in Both Acidic and Alkaline Media

Lijie Zhang, Haeseong Jang, Yan Wang, Zijian Li, Wei Zhang, Min Gyu Kim, Dongjiang Yang, Shangguo Liu, Xien Liu,* and Jaephil Cho**

Dr. Lijie Zhang, Zijian Li, Dr. Shangguo Liu, Prof. Xien Liu
State Key Laboratory Based of Eco-Chemical Engineering, College of Chemical Engineering, Qingdao University of Science and Technology, Qingdao 266042, P. R. China
E-mail: liusg@qust.edu.cn; liuxien@qust.edu.cn
Dr. Haeseong Jang, Prof. Jaephil Cho
Department of Energy Engineering and School of Energy and Chemical Engineering, Ulsan National Institute of Science and Technology (UNIST) Ulsan 689-798, South Korea
E-mail: jpcho@unist.ac.kr
Dr. Yan Wang, Prof Wei Zhang
Electron Microscopy Center, and Key Laboratory of Automobile Materials MOE, Jilin University, Changchun 130012. China
E-mail: weizhang@jlu.edu.cn
Prof. Min Gyu Kim
Beamline Research Division, Pohang Accelerator Laboratory (PAL), Pohang 790-784, Korea
Prof. Dongjiang Yang,
School of Environmental Science and Engineering, State Key Laboratory of Bio-fibers and Eco-textiles, Collaborative Innovation Center of Marine Biobased Fibers and Ecological textiles, Institute of Marine Biobased Materials, Qingdao University, P. R. China.

This PDF file includes:

Experimental Sections, DFT calculations, Statistical Analysis

Figure S1 to S32, Table S1 to S5

Experimental Sections

Materials. Metal sources $RuCl_3 \cdot xH_2O$ and sodium alginate were purchased from Aladdin. Ar gases were supplied in cylinders by Heli factory with 99.999% purity. Nafion® perfluorinated resin solution containing 5% Nafion® was purchased from Sigma-Aldrich. Ultrapure water with resistivity > 18 MΩ cm^{-1} was used.

Preparation of Ru_{SA+NP}/DC. The synthesis process of Ru_{SA+NP}/DC is as follows. Firstly, 0.10 g $RuCl_3 \cdot xH_2O$ powder was dissolved in 100 mL mixed water/ethanol (V_{water}:$V_{ethanol}$ = 3:2) solution. Secondly, the $RuCl_3$ solution was slowly added into 100 mL 1 wt.% sodium alginate solution with stirring, obtaining Ru-alginate hydrogels. Then, the as-prepared hydrogels were dehydrated via a freeze-drying process to obtain three dimensional (3D) Ru-alginate aerogels. In this step, each Ru^{3+} ion is stabilized and confined in the "egg-box" structure by coordinating with the –COO– groups in alginate macromolecules, avoiding their migration in initial annealing stage.[1] Subsequently, the Ru-alginate aerogels aerogels were placed in a tube furnace and heated in Ar atmosphere from room temperature to 250 °C where they were treated for 1 h, and subsequently heated to 1000 °C and treated for 1.5 h. During this process, the alginate macromolecules were decomposed, generating abundant H_2O and CO_2.[2] The carbon skeleton was converted to highly porous defective carbon substrate *via* carbon etching reaction[3], providing rich anchoring sites for SAs. Meanwhile, the Ru^{3+} ions were reduced *in situ* by the generated carbon, converting to Ru NPs as well as SAs through agglomeration and atomization processes.[4] Finally, the as-obtained products were leached in 2 M HCl to remove natural impurities (such as Na^+) and dried in vacuum, obtaining Ru_{SA+NP}/DC. Notably,

this synthesis strategy is of high potential to be scaled up to an industrial level owing to the cheap resources and simple production process.

For comparison, using the same method, Ru_{SA+NP}/DC samples at different temperatures (900 and 1100°C) are synthesized by only changing the carbonization temperatures, denoted as Ru_{SA+NP}/DC (900°C) and Ru_{SA+NP}/DC (1100°C). Ru_{SA+NP}/DC samples with different Ru contents were prepared by only changing the additive amounts of $RuCl_3·xH_2O$ (0.05 and 0.15 g), denoted as Ru_{SA+NP}/DC (0.05 g) and Ru_{SA+NP}/DC (0.15 g).

Preparation of Ru_{SA}/DC. The synthesis process of Ru_{SA}/DC is the same as that of Ru_{SA+NP}/DC except that 10 mL 2 M HCl and 0.010 g $RuCl_3·xH_2O$ was dissolved in 100 mL mixed water/ethanol (V_{water}:$V_{ethanol}$ = 3:2) solution. Meanwhile, sample with lower Ru content was synthesized by changing the additive amount of $RuCl_3·xH_2O$ to 0.005 g, denoted as Ru_{SA}/DC (0.005).

Preparation of DC. The synthesis process of DC is the same as that of Ru_{SA+NP}/DC except that no addition of $RuCl_3·xH_2O$.

Characterizations

The morphologies of the samples were characterized by field emission scanning electron microscopy (FESEM; SU8020). Transmission electron microscopy were collected from FEI Tecnai G20 and probe-corrected JEOL ARM200F with acceleration voltages of 120 and 80 kV, respectively. The chemical composition was investigated by X-ray photoelectron spectroscopy (XPS) using an ESCALab250 electron spectrometer (Thermo Scientific Corporation) with mono-chromatic 150 W Al

Kα radiation. The phase structures were characterized with X-ray diffraction (XRD, DX2700, China) operating with Cu Kα radiation (l=1.5418 Å) at a scan rate (2θ) of 1° min^{-1} with the accelerating voltage of 40 kV. For X-ray absorption near edge structure (XANES) and extended X-ray absorption fine structure (EXAFS), the data collected at the Pohang Light Source (PLS-II) in Korea.

The calculation of electrochemical active surface area (ECSA)

In this work, we use electrochemical double layer capacitances (C_{dl}) to characterize electrochemical active surface area (ECSA). The C_{dl} was measured by cyclic voltammograms in a potential region of 0.05 V to 0.15 V *vs.* RHE, where there is no Faradic current. The C_{dl} is estimated by plotting the ΔJ/2 at 0.1 V *vs.* RHE against the scan rates, where the slope is C_{dl}. The ECSA of the catalyst layer can be calculated as Equation (1):

$$ECSA = \frac{C_{dl}}{C_S} \qquad (1)$$

Cs is the specific capacitance value for a flat standard with 1 cm^2 of real surface area. The *Cs* values were chosen as 0.035 mF cm^{-2} in 0.5 M H_2SO_4 and 0.04 mF cm^{-2} in 1 M KOH.[5]

The calculation of turnover frequency (TOF)

The TOF is calculated by the following Equation (2)~(4), according to previous literature[6],

$$TOF = \frac{N_{H_2\ per\ unit\ area}}{N_{active\ site\ per\ unit\ area}} \qquad (2)$$

$$N_{H_2\ per\ unit\ area} = \frac{j}{2F} \qquad (3)$$

$$N_{active\ site\ per\ unit\ area} = N_{metal\ atom} = \frac{m_{metal}}{M} \qquad (4)$$

The *F* is Faraday constant, the *j* is current density, and the *M* is atomic mass of Ru/Pt.

DFT Calculations

The DFT calculations were performed with VASP.[7] The Perdew-Burke-Ernzerhof (PBE) functional was employed to treat the exchange-correlation interactions[8]. For structure relaxation and the calculation of electronic properties, the plane wave basis set with a kinetic energy cutoff of 500 eV and the energy convergence criterion of 10^{-5} eV and a (2×2×1) Monkhorst-Pack k-point sampling was employed. The graphene monolayers were modeled using a (6 × 6) supercell for Ru_{SA}@DC-1 and Ru_{SA}@DC-2, and were modeled using a (8 × 8) supercell for Ru_{NP}@DC-1, Ru_{NP}@DC-2 and Pt_{NP}@DC-1. A vacuum region of 15 Å was employed to prevent the interaction between neighboring structures along the *z* axis. H_2O and H_2 were calculated in boxes of 15 Å×15 Å×15 Å with the gamma point. The CI-NEB method was adopted to search the minimum energy paths of H_2O dissociation reaction[9, 10]. Six images were used for CI-NEB calculations. The free energy diagrams for HER were calculated with reference to the computational hydrogen electrode.[11] The free energy of gas phase and adsorbed species can be obtained from the following equation (5):

$$G = E_{elec} + ZPE - TS \qquad (5)$$

where E_{elec} is the electronic energy. Details of zero point energy (ZPE) and entropy contribution entropies (-TS) correction of gas phase, and adsorbed species are shown in the following table.

Species	H_2	H*
ZPE	0.27	0.17
-TS	-0.41	0

The *d* band center (ε) was calculated based on the following equation (6):

$$\varepsilon = \frac{\int_{-\infty}^{\infty} \rho(x)xdx}{\int_{-\infty}^{\infty} \rho(x)dx} \qquad (6)$$

where $\rho(x)$ is the PDOS at the energy of x.

Statistical Analysis

1. The high-resolution XPS spectra were fitted by the XPSPEAK41 software.

2. To calculate the electrochemically active surface areas (ECSAs) of samples, a linear trend was obtained by plotting the difference between the anodic and cathodic current densities against the scan rate at 0.15 V *vs*. RHE by using the Origin software. The R^2 (statistics) values of the fitting lines for all the samples are above 0.998.

3. The EXAFS spectra were fitted by the Artemis software. The R factor is within 0.003.

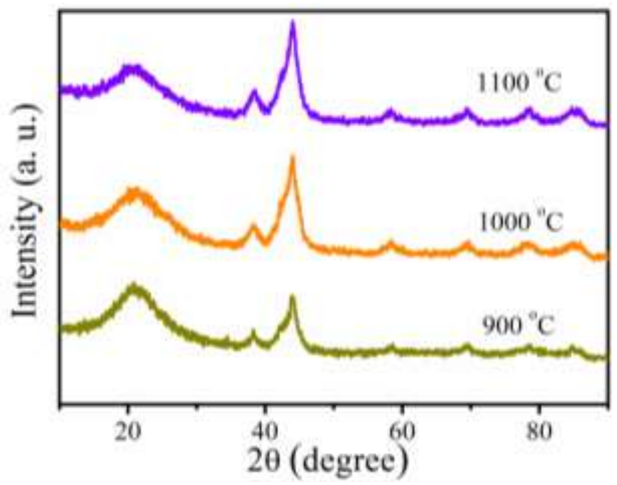

Figure S1. XRD patterns of Ru_{SA+NP}/DC (900 °C), Ru_{SA+NP}/DC and Ru_{SA+NP}/DC (1100 °C).

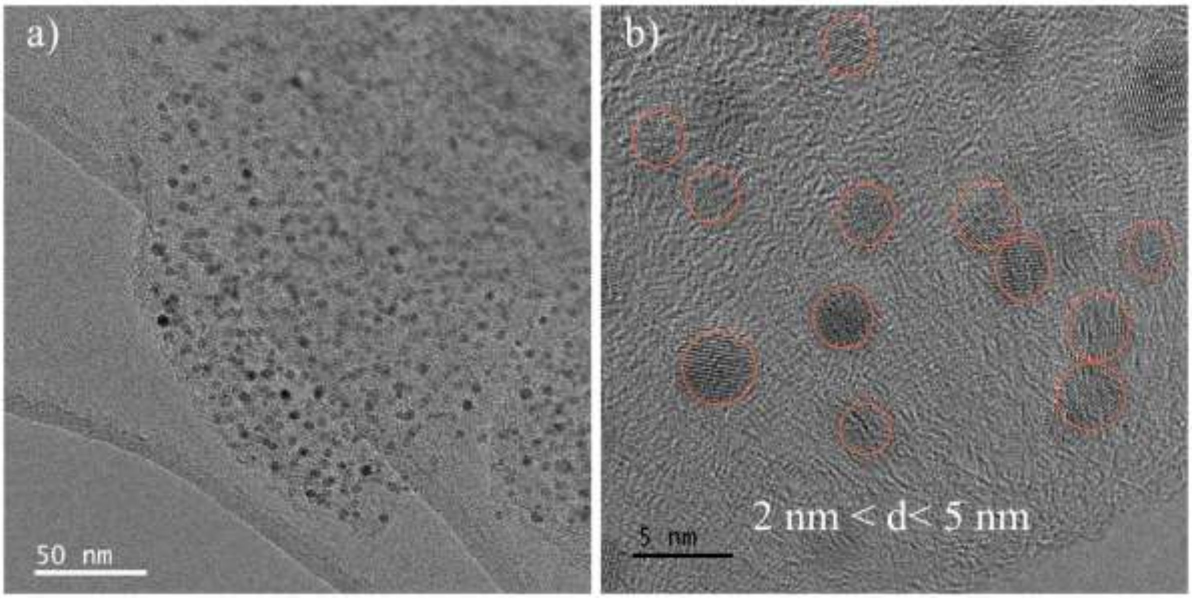

Figure S2. (a) TEM and (b) HRTEM images of the as-synthesized Ru_{SA+NP}/DC.

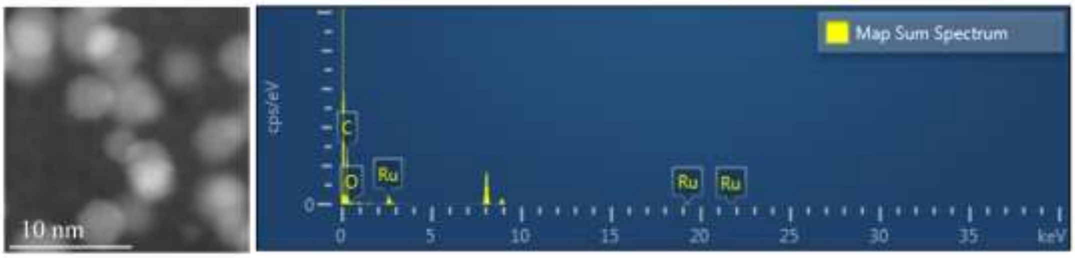

Figure S3. EDS analysis of Ru_{SA+NP}/DC.

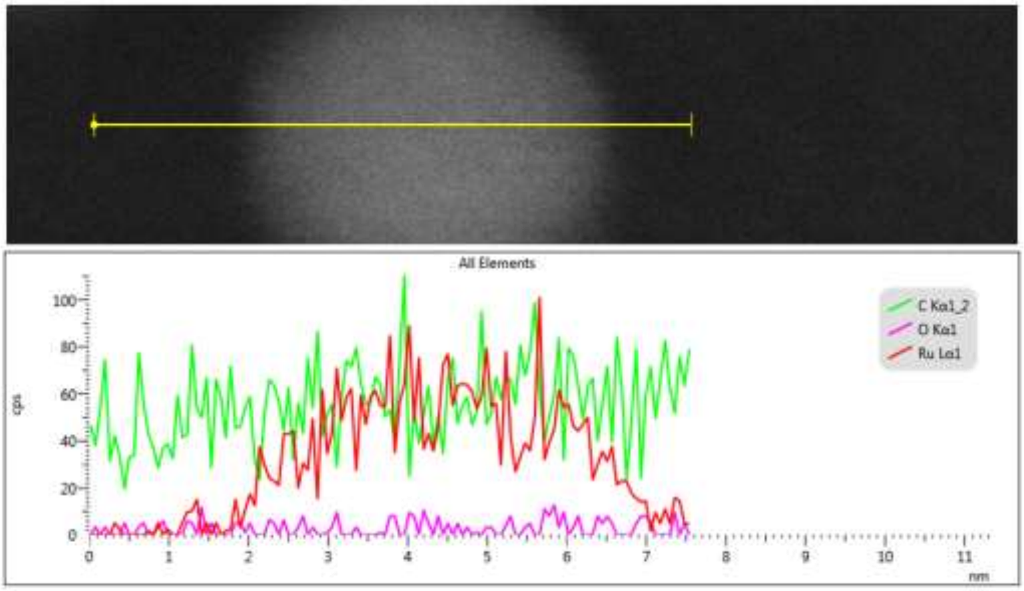

Figure S4. EDS line scanning of Ru_{SA+NP}/DC and the linear distributions of Ru, C, O.

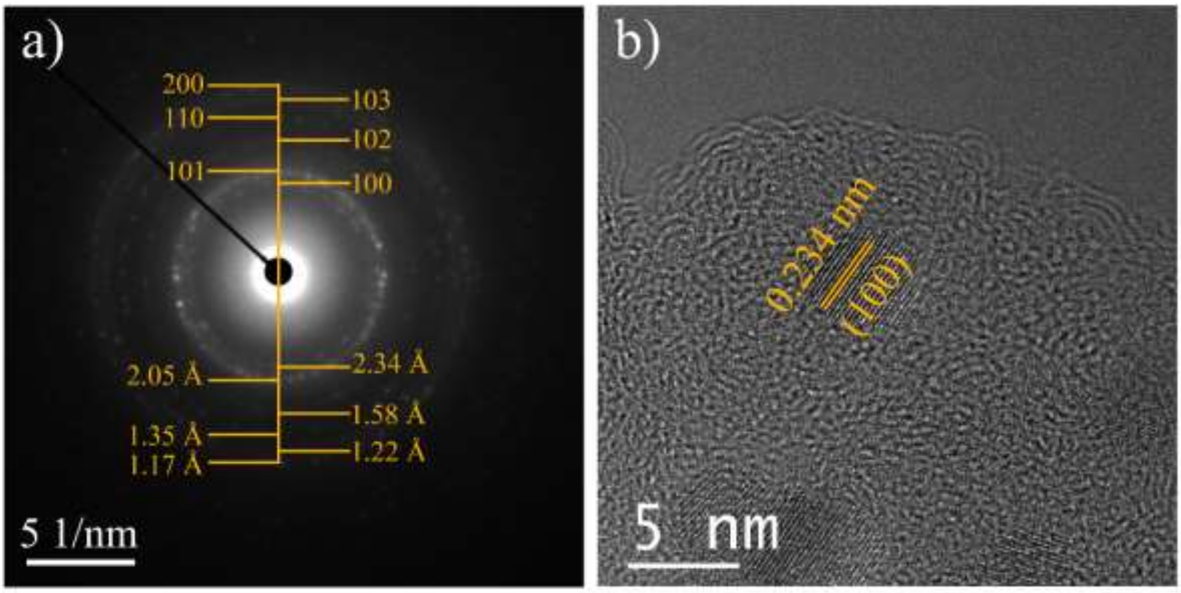

Figure S5. (a) The selected area electron diffraction (SAED) pattern and (b) HRTEM image of the as-synthesized Ru_{SA+NP}/DC.

The SAED pattern in Figure S5a can be well indexed to the (100), (101), (101), (110), (102), (103) and (200) planes of hexagonal Ru, repectively. The lattice fringes with interplanar spacings of 2.34 Å is ascribed to the (100) facets of hexagonal Ru (Figure S5b).

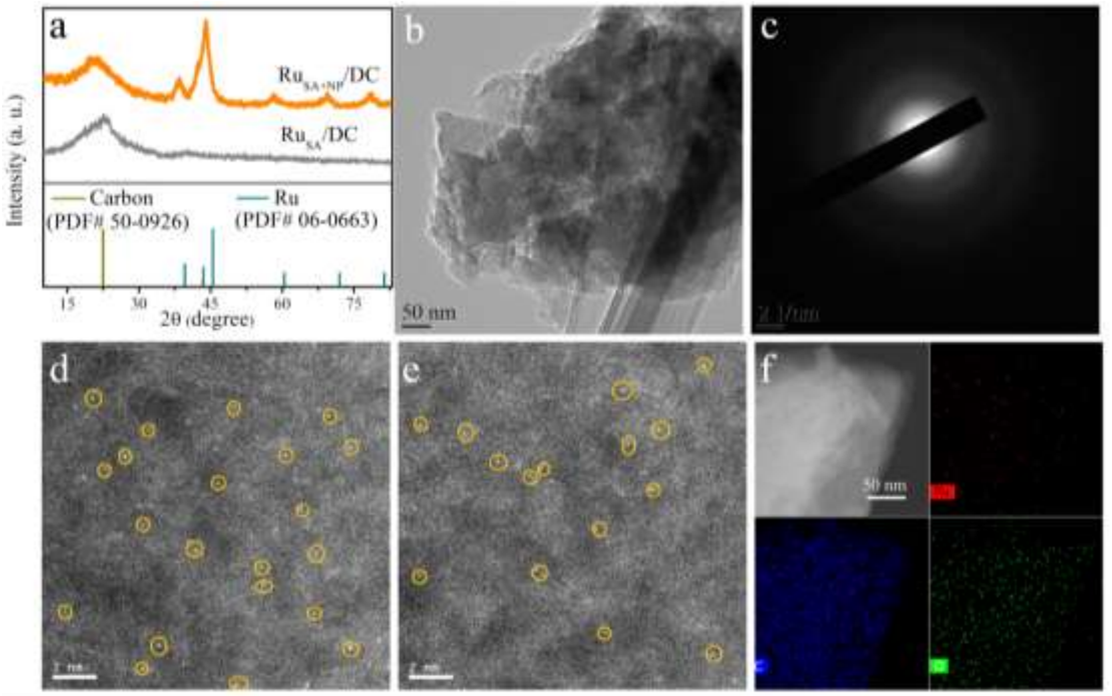

Figure S6. (a) The XRD patterns of as-synthesized Ru_{SA+NP}/DC and Ru_{SA}/DC. (b-c) TEM and corresponding selected area electron diffraction (SAED) of Ru_{SA}/DC. (d-e) HAADF-STEM images of Ru_{SA}/DC in different areas. (f) The corresponding EDS mapping images of Ru_{SA}/DC.

As presented in the XRD pattern, no diffraction peaks of crystalline Ru are detected. From the TEM image, no Ru NPs are observed. The corresponding selected area electron diffraction (SAED) displays no obvious diffraction rings and spots, indicating not detectable Ru. HAADF-STEM reveals abundant atomically dispersed bright spots are distributed in the carbon, ascribing to atomic Ru (highlighted by yellow circles). The corresponding mapping images indicate that Ru, C, and O were distributed homogeneously over the entire architecture.

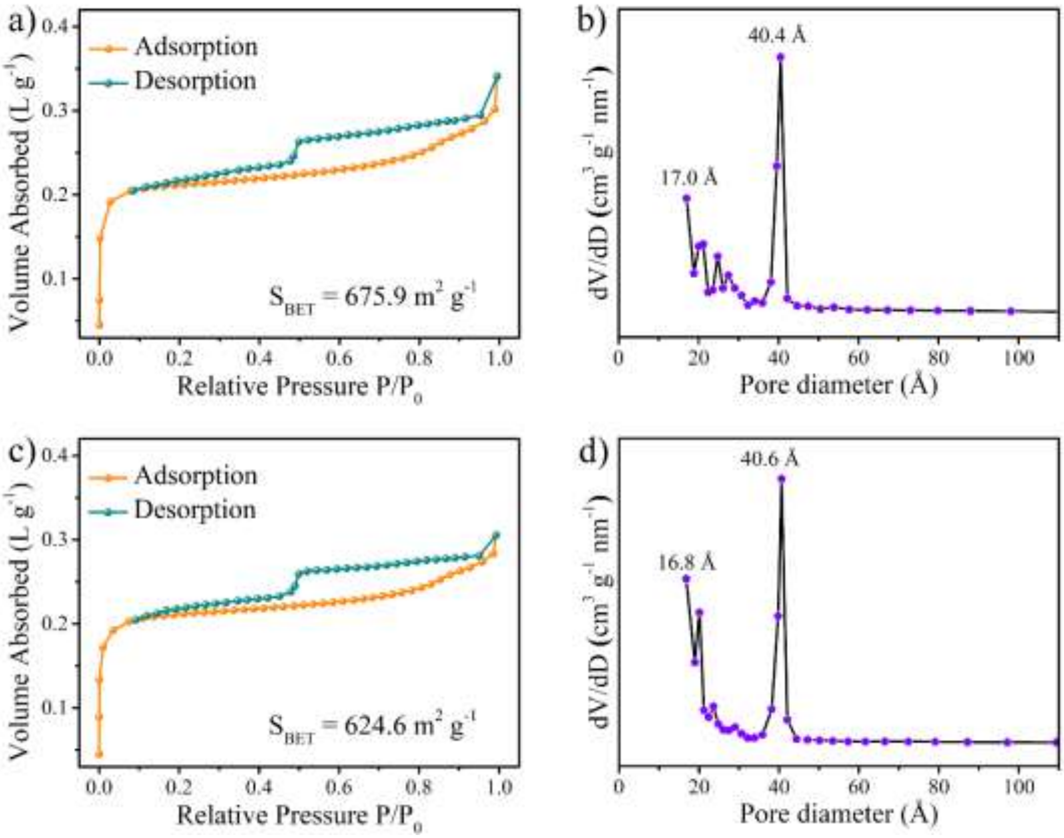

Figure S7. (a-b) N_2 adsorption/desorption isotherms (a) and corresponding BJH pore diameter (b) of Ru_{SA+NP}/DC (900 °C). (c-d) N_2 adsorption/desorption isotherms (a) and corresponding BJH pore diameter (b) of Ru_{SA+NP}/DC (1100 °C).

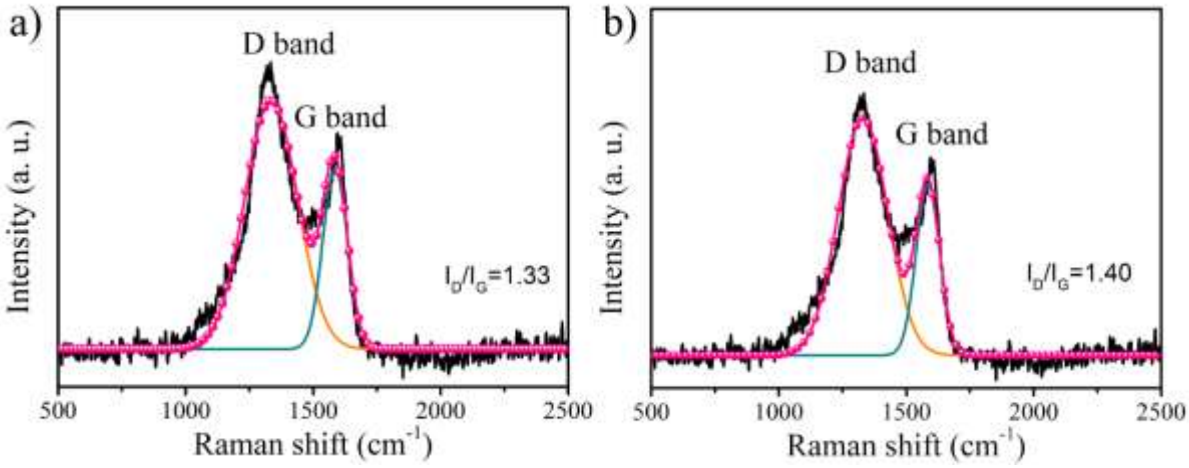

Figure S8. Raman spectra of Ru_{SA+NP}/DC synthesized at 900°C (a) and 1100 °C (b).

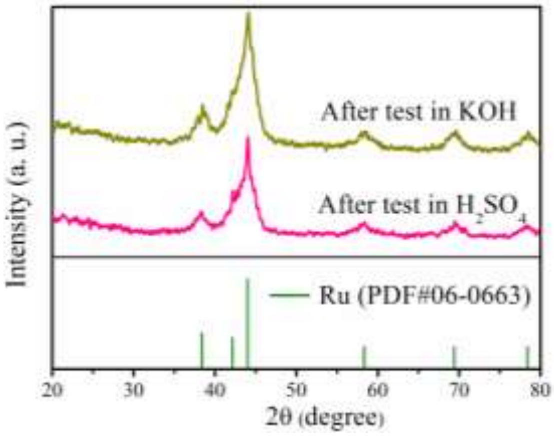

Figure S9. XRD patterns of Ru_{SA+NP}/DC after HER test in 0.5 M H_2SO_4 and 1 M KOH.

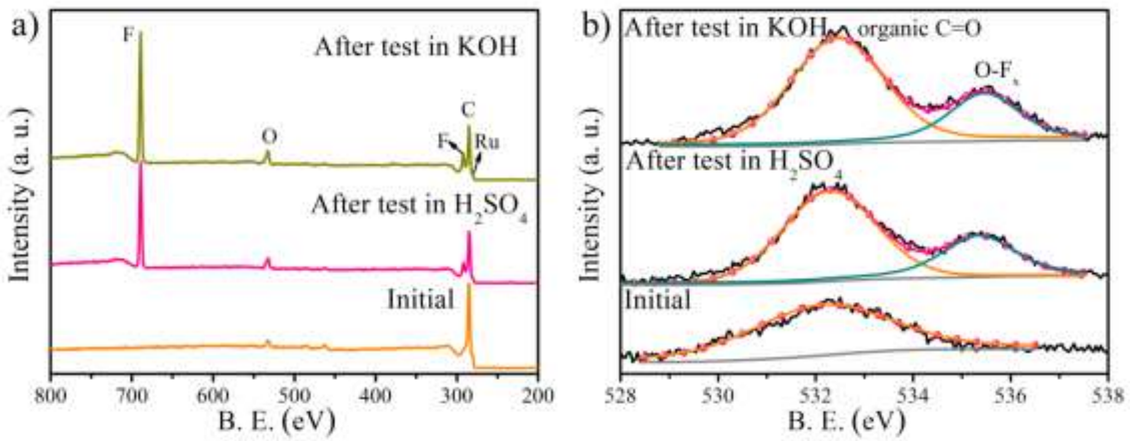

Figure S10. The full scan spectrum (a) and high-resolution XPS of O 1s for initial Ru_{SA+NP}/DC samples, Ru_{SA+NP}/DC after test in H_2SO_4, and Ru_{SA+NP}/DC after test in KOH.

The presence of F element and –O-F_x groups in the samples after test is attributed to the use of 5 wt % Nafion solutions in the preparation of working electrode. The main peak in O 1s is attributed to the organic C=O species, generating by the carbonization of sodium alginate macromolecular.

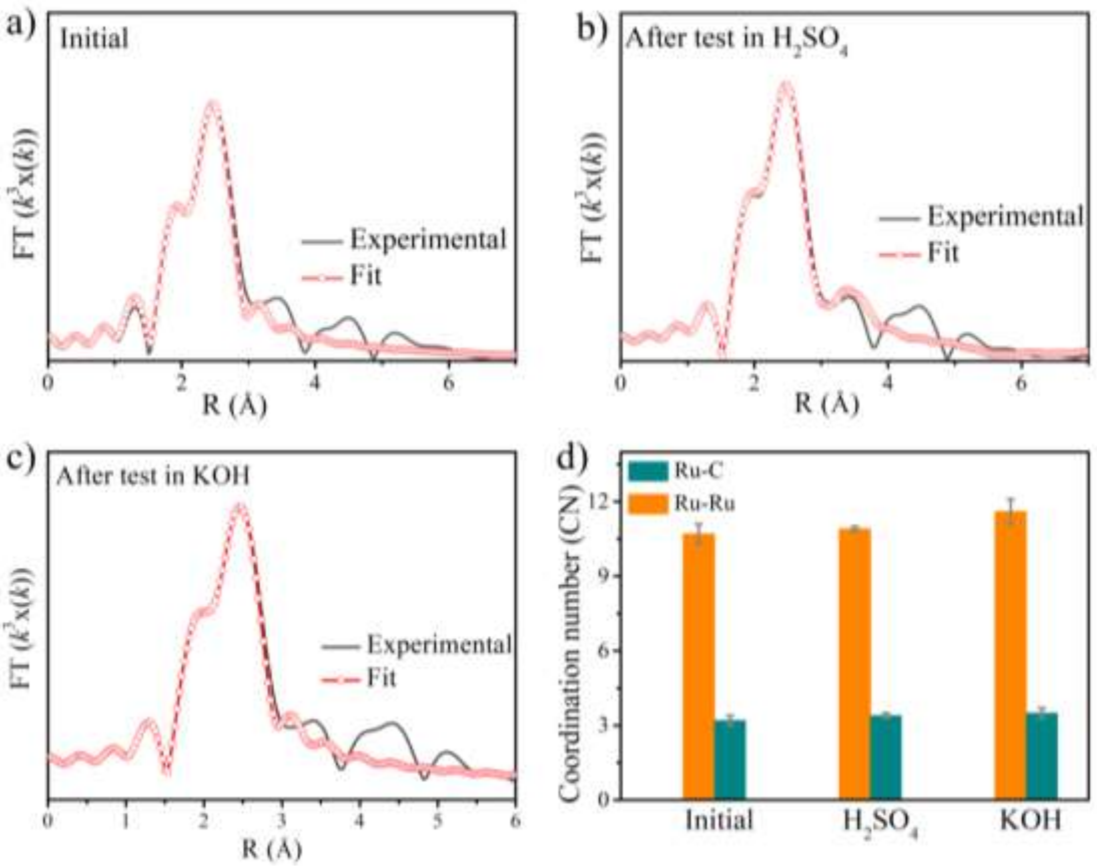

Figure S11. The experimental and fitting curves of FT-EXAFS spectra for (a) initial Ru_{SA+NP}/DC, (b) Ru_{SA+NP}/DC after HER test in H_2SO_4, (c) Ru_{SA+NP}/DC after HER test in KOH and (d) the corresponding coordination numbers.

All the FT-EXAFS spectra for the samples before and after HER test can be well fitted with Ru-Ru and Ru-C paths, where the Ru-C paths are ascribed to the Ru SAs in catalysts. According to the fitting parameters given in Tables S3, the coordination numbers of Ru-C are in the range of 3-4 for both samples before and after HER test. That is to say that Ru SAs are coordinated with the C atoms in DC, and the mainly structures are identified as RuC_3 and RuC_4 moieties.

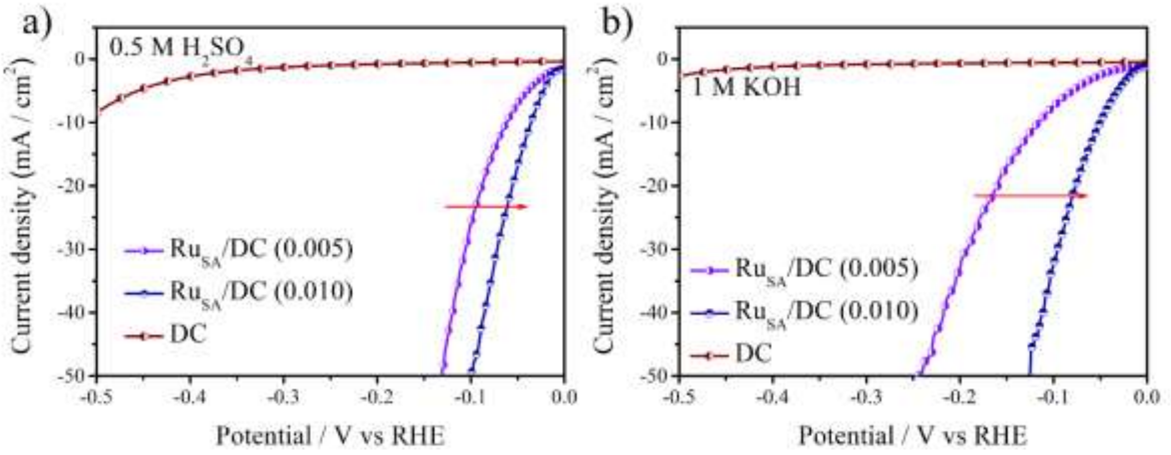

Figure S12. Comparison of HER polarization curves of Ru_{SA}/DC (0.005), Ru_{SA}/DC (0.010), and DC in (a) 0.5 M H_2SO_4 and (b) 1 M KOH.

It can be observed that the DC exhibit ultrahigh η_{10} values (> 500 mV) in both acidic and alkaline media, demonstrating the DC substrate contribute little to HER activities. However, both acidic and alkaline HER activities are dramatically enhanced when the Ru atoms anchored on DC, indicating Ru sites is the active sites and responsible for HER activities. Obviously, the HER activities are improved with the increase of Ru content, as demonstrated by the superior HER activity of Ru_{SA}/DC (0.010) to Ru_{SA}/DC (0.005).

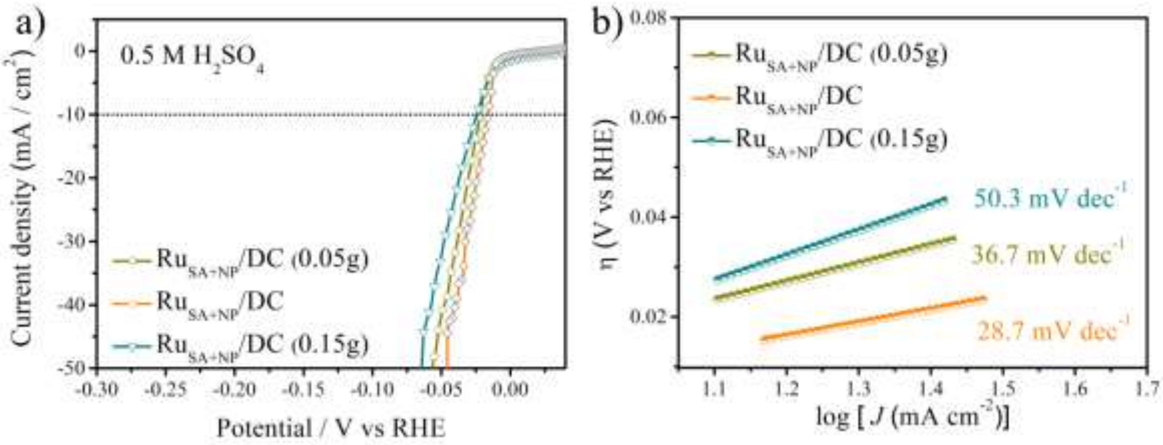

Figure S13. Comparison of HER polarization curves and (a) and corresponding Tafel slopes (b) in 0.5 M H_2SO_4 for Ru_{SA+NP}/DC samples synthesized at 1000°C with different additive amounts of $RuCl_3$.

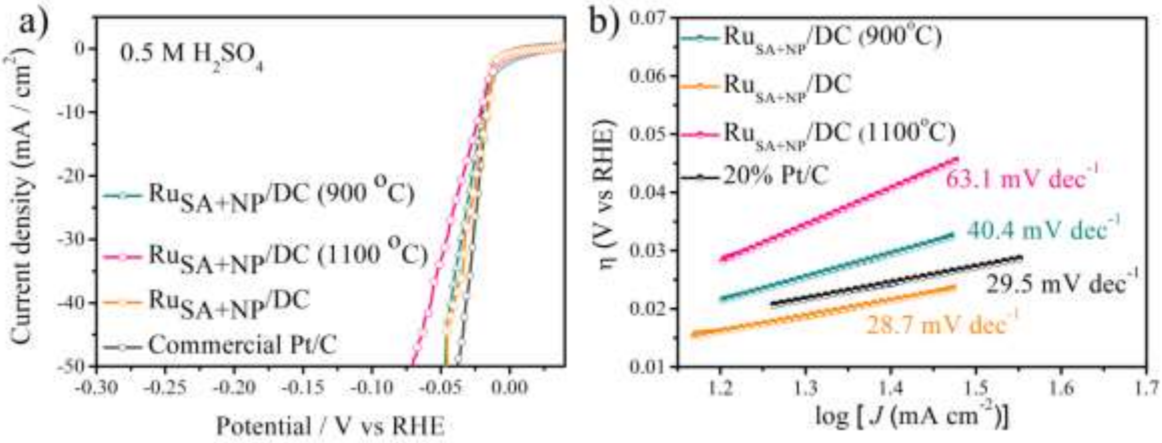

Figure S14. Comparison of HER polarization curves and (a) and corresponding Tafel slopes (b) in 0.5 M H_2SO_4 for commercial Pt/C and Ru_{SA+NP}/DC samples with 0.10 g $RuCl_3$ synthesized at different temperatures.

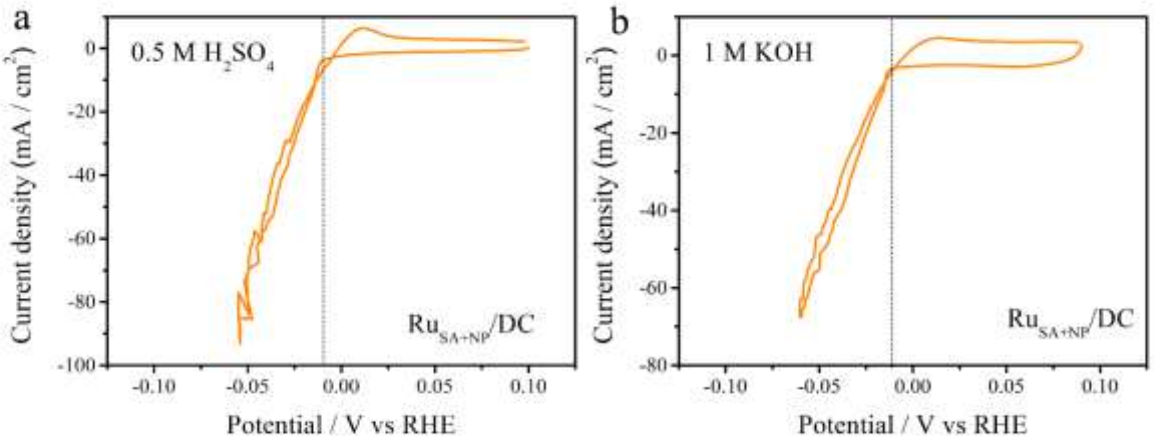

Figure S15. The CV profiles of Ru_{SA+NP}/DC in (a) 0.5 M H_2SO_4 and (b) 1 M KOH.

In order to observe the forward and backward scans, the CV curves for Ru_{SA+NP}/DC are tested under the same conditions. As shown in Figure S15, the obvious reduction reaction occurs at about −9 and −10 mV in 0.5 M H_2SO_4 and 1 M KOH, respectively. Beyond this potential, the current density increases dramatically.

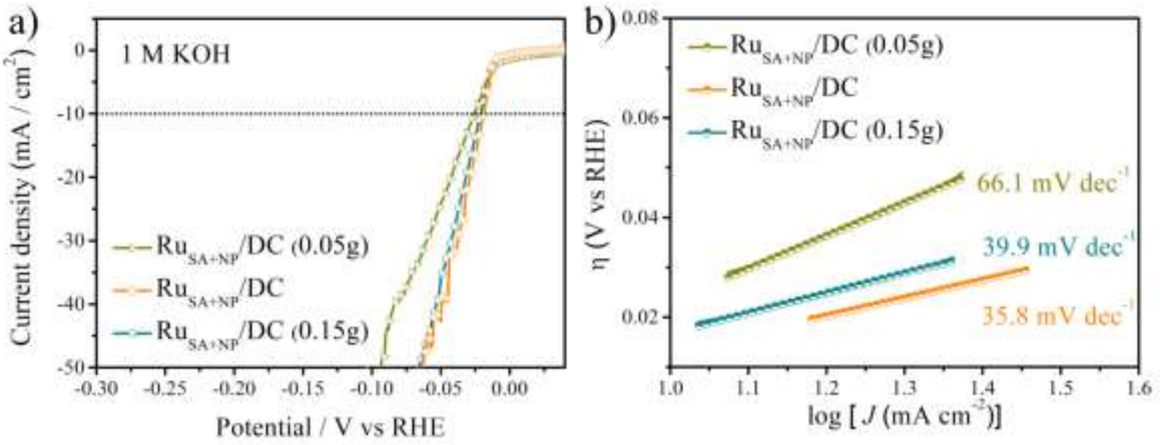

Figure S16. Comparison of HER polarization curves and (a) and corresponding Tafel slopes (b) in 1 M KOH for Ru_{SA+NP}/DC samples synthesized at 1000°C with different additive amounts of $RuCl_3$.

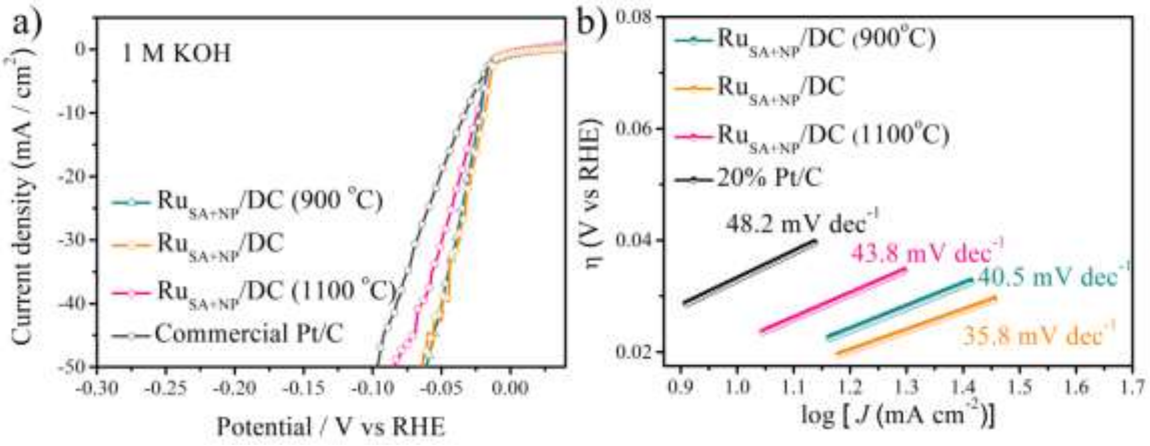

Figure S17. Comparison of HER polarization curves and (a) and corresponding Tafel slopes (b) in 1 M KOH for commercial Pt/C and Ru_{SA+NP}/DC samples with 0.10 g $RuCl_3$ synthesized at different temperatures.

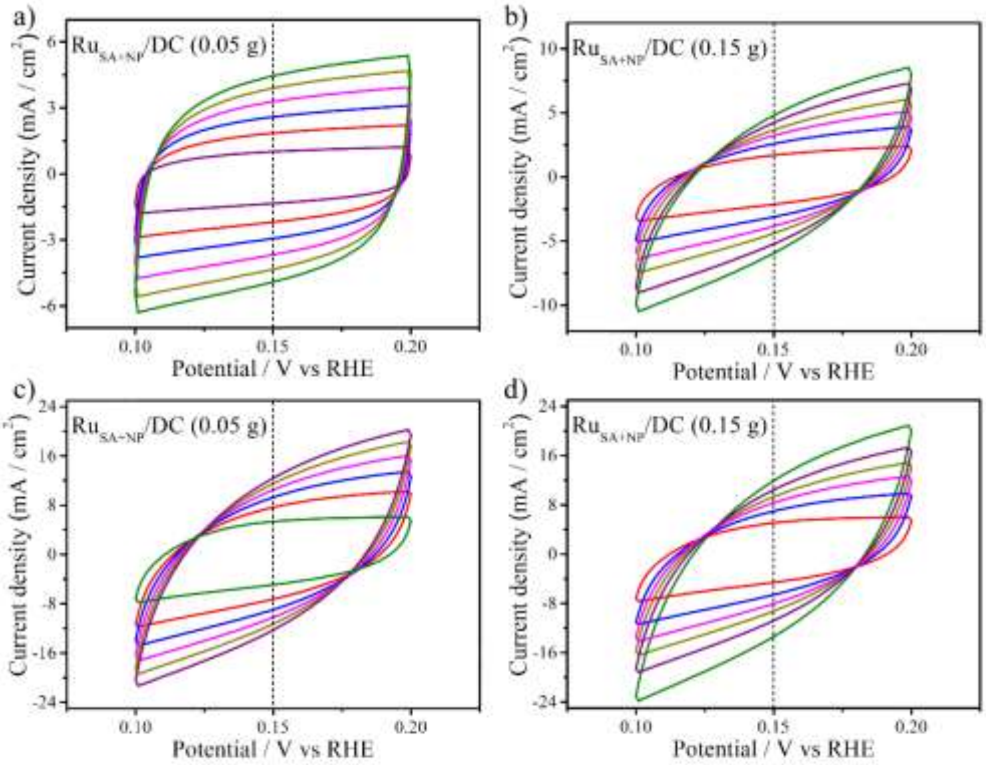

Figure S18. CV curves of Ru_{SA+NP}/DC samples synthesized at 1000°C with different additive amounts of $RuCl_3$ at various scan rates (20, 40, 60, 80, 100, and 120 mV s^{-1}) in 0.5 M H_2SO_4 (a-b) and 1 M KOH (c-d).

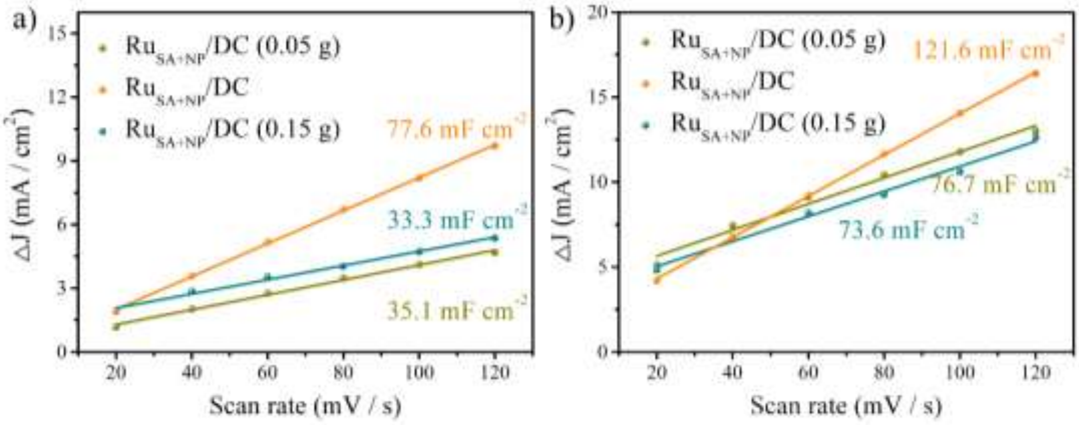

Figure S19. Comparison of ECSAs for Ru_{SA+NP}/DC samples synthesized at 1000°C with different amounts of $RuCl_3$ in 0.5 M H_2SO_4 (a) and 1 M KOH (b).

Based on the calculated C_{dl} and Equation (1), the ECSAs of Ru_{SA+NP}/DC (0.05g), Ru_{SA+NP}/DC, Ru_{SA+NP}/DC (0.15g) in 0.5 M H_2SO_4 are 1002.9, 2217.1 and 945.7 cm^2, respectively. The ECSAs of Ru_{SA+NP}/DC (0.05g), Ru_{SA+NP}/DC, Ru_{SA+NP}/DC (0.15g) in 1 M KOH are 1917.5, 3040.0 and 1840.0 cm^2, respectively.

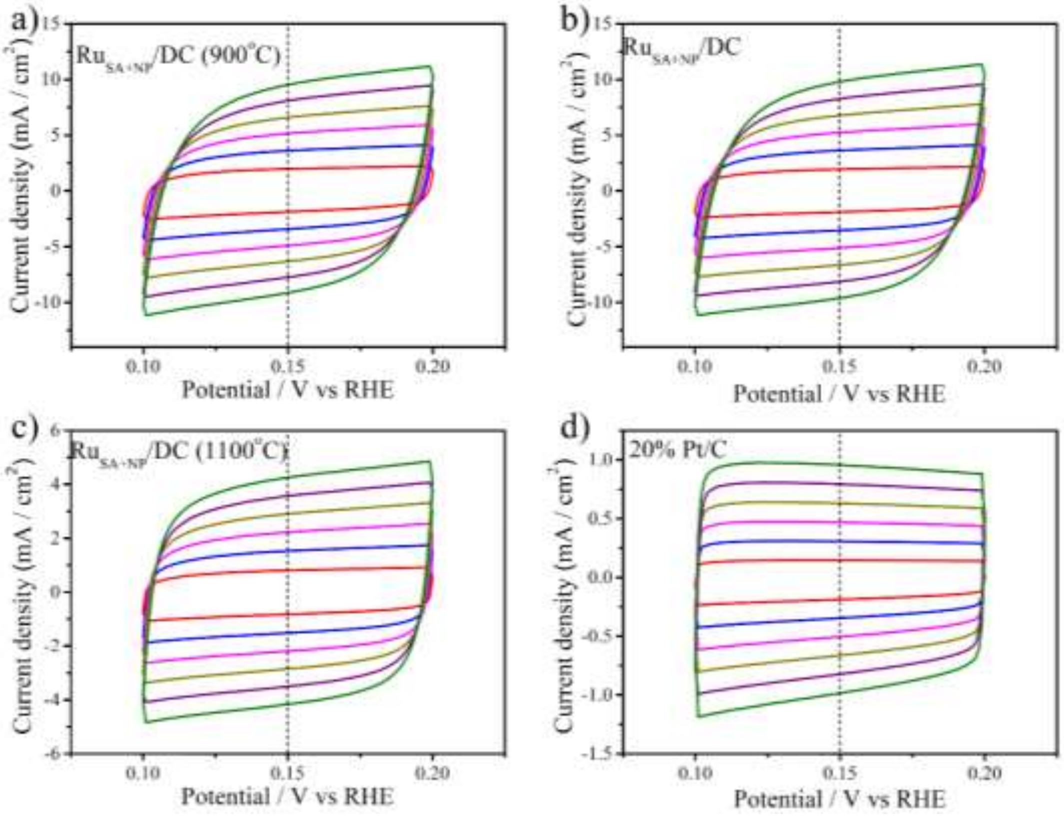

Figure S20. CV curves of Ru$_{SA+NP}$/DC samples with 0.10 g $RuCl_3$ synthesized at 900°C (a), 1000°C (b), 1100°C (c) and (d) the commercial Pt/C at various scan rates (20, 40, 60, 80, 100, and 120 mV s^{-1}) in 0.5 M H_2SO_4.

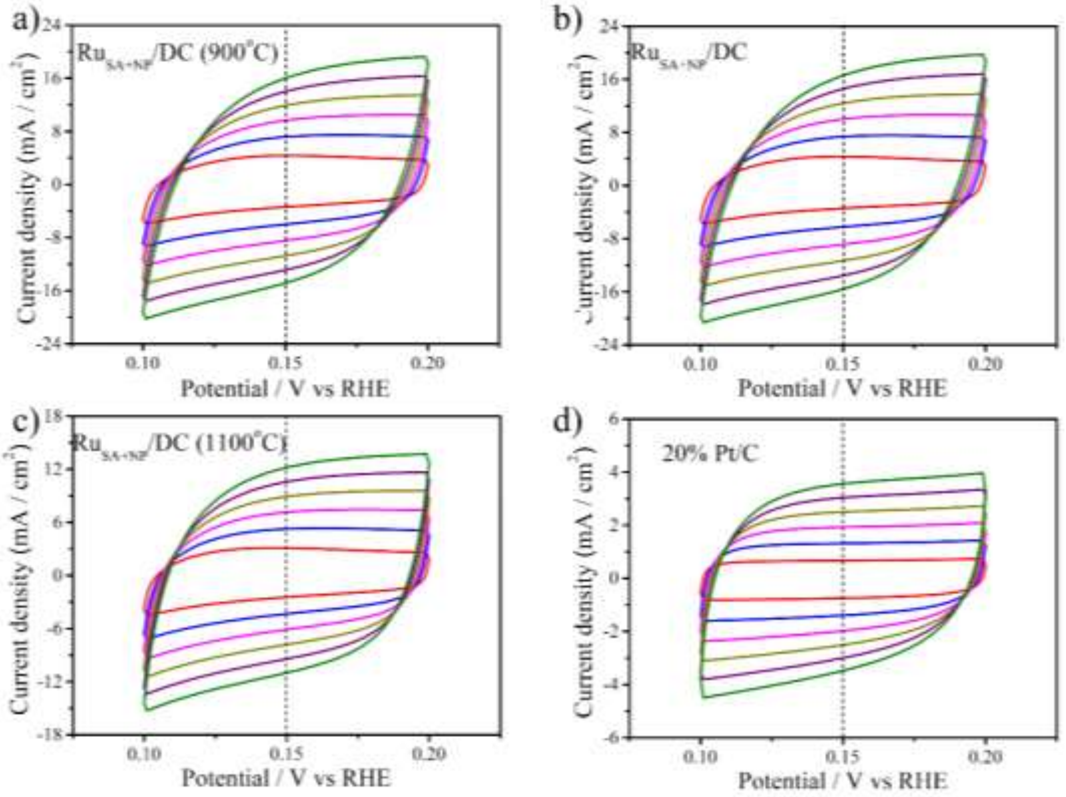

Figure S21. CV curves of Ru_{SA+NP}/DC samples with 0.10 g $RuCl_3$ synthesized at 900°C (a), 1000°C (b), 1100°C (c) and (d) the commercial Pt/C at various scan rates (20, 40, 60, 80, 100, and 120 mV s^{-1}) in 1 M KOH.

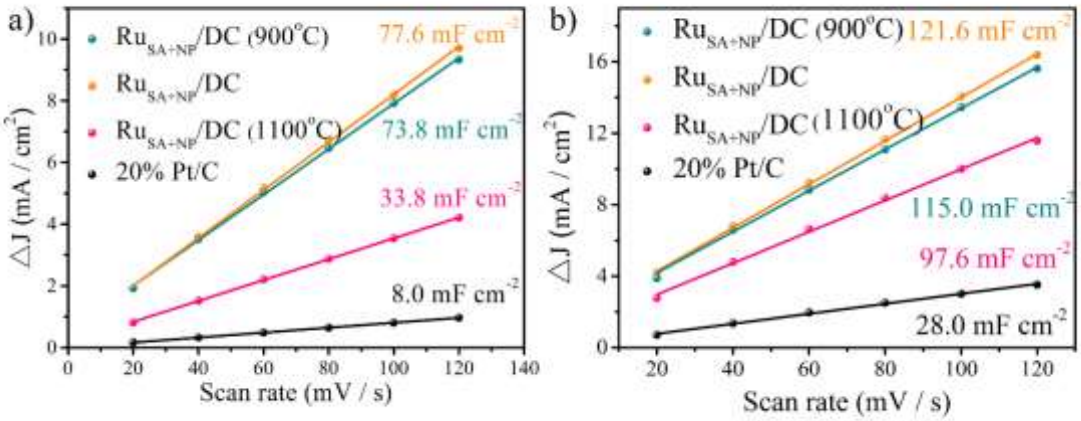

Figure S22. Comparison of ECSAs for commercial Pt/C and Ru$_{SA+NP}$/DC samples synthesized at different temperatures at 0.5 M H_2SO_4 (a) and 1 M KOH (b).

Based on the calculated C_{dl} and Equation (1), the ECSAs of Ru$_{SA+NP}$/DC (900°C), Ru$_{SA+NP}$/DC, Ru$_{SA+NP}$/DC (1100°C) and 20% Pt/C in 0.5 M H_2SO_4 are 2108.6, 2217.1, 965.7 and 228.6 cm^2, respectively. The ECSAs of Ru$_{SA+NP}$/DC (900°C), Ru$_{SA+NP}$/DC, Ru$_{SA+NP}$/DC (1100°C) and 20% Pt/C in 1 M KOH are 2875.0, 3040.0, 2440 and 700.0 cm^2, respectively.

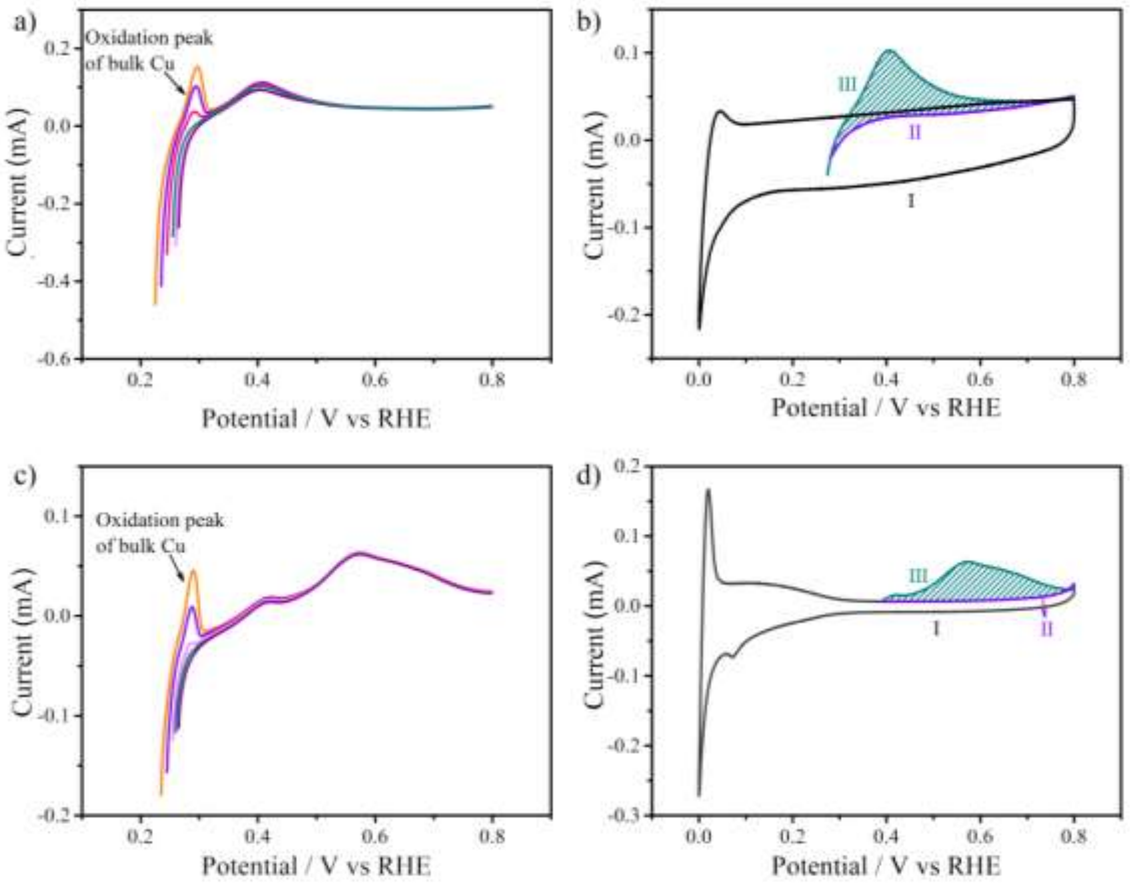

Figure S23. Cu UPD of (a) Ru_{SA+NP}/DC and (c) commercial Pt/C in 0.5 M H_2SO_4 + 20 mM $CuSO_4$ solution. The electrode was polarized from 0.265, 0.260, 0.255, 0.245, 0.235 and 0.225 V to form the UPD layers, respectively. Cu UPD of (b) Ru_{SA+NP}/DC and (d) commercial Pt/C in 0.5 M H_2SO_4 in the (I, II) absence and (III) presence of 20 mM $CuSO_4$. For II and III, the electrode of Ru_{SA+NP}/DC and Pt/C was polarized at 0.255 and 0.260 V, respectively.

The underpotential deposition (UPD) method was used to quantitatively analyse the number of active sites of Ru_{SA+NP}/DC and commercial Pt/C. As shown in Figure S23a, under the polarization potentials of 0.265, 0.260 and 0.255 V, only one oxidation peak is observed, which belongs to the underpotentially deposited mono- or submonolayer Cu. When the potential was decreased to 0.245, 0.235 and 0.225 V, another oxidation peak located at

relatively lower potential appears, which can be attributed to the oxidation of bulk Cu. copper. To obtain a monolayer copper, 0.255 V was selected in the following test of Ru_{SA+NP}/DC (Figure S23b). The commercial Pt/C was also investigated using the same method (Figure S23c-d). Then, the number of active sites (*n*) can be qualified based on the UPD Cu stripping charges (Q_{Cu}, $Cu_{upd} \rightarrow Cu^{2+} + 2e^{-}$) with the equation of $n=Q_{Cu}/2F$ (where F is the Faraday constant). The electrode area is 0.0706 cm^2. After calculation, the active site density of Ru_{SA+NP}/DC is 1.13×10^{14} sites per cm^2, whereas it is 9.94×10^{13} sites per cm^2 for that of Pt/C. Thus, the Ru_{SA+NP}/DC possesses more active sites than Pt/C.

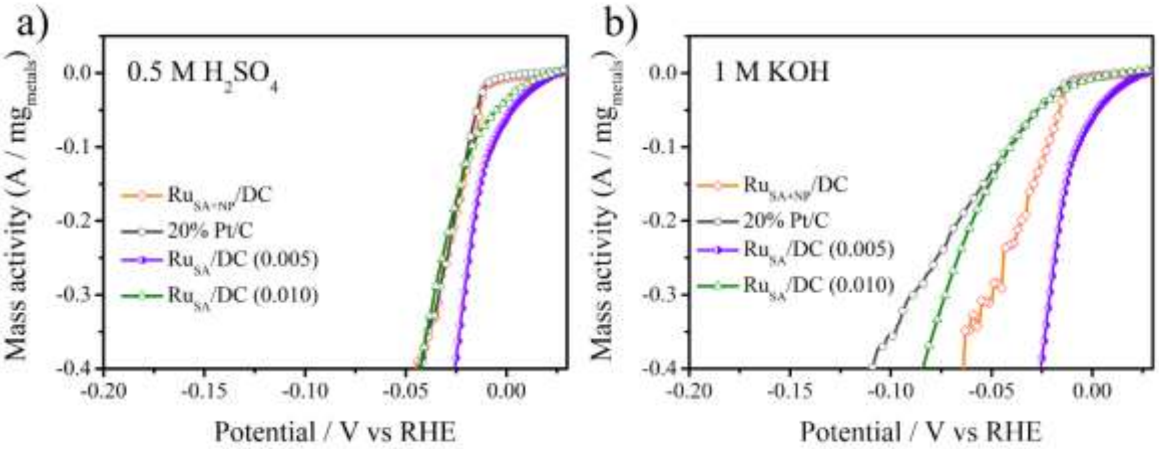

Figure S24. Mass activities of Ru_{SA+NP}/DC, Ru_{SA}/DC (0.005), Ru_{SA}/DC (0.010) and Pt/C in (a) 0.5 M H_2SO_4 and (b) 1 M KOH.

The mass activities of Ru_{SA}/DC in both acidic and alkaline media are improved with the increase of Ru loading, and become comparable or even better than those of Ru_{SA+NP}/DC when the Ru SAs content achieves the highest. These results demonstrate the high atom utilization of single atom electrocatalysts.

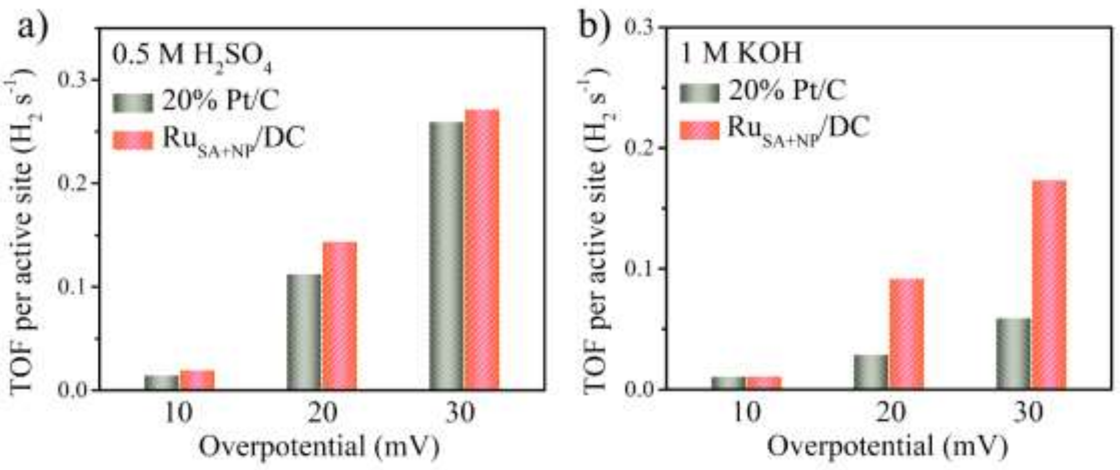

Figure S25. Comparison of TOF values for Ru_{SA+NP}/DC and Pt/C in (a) 0.5 M H_2SO_4 and (b) 1 M KOH at the overpotentials of 10, 20 and 30 mV.

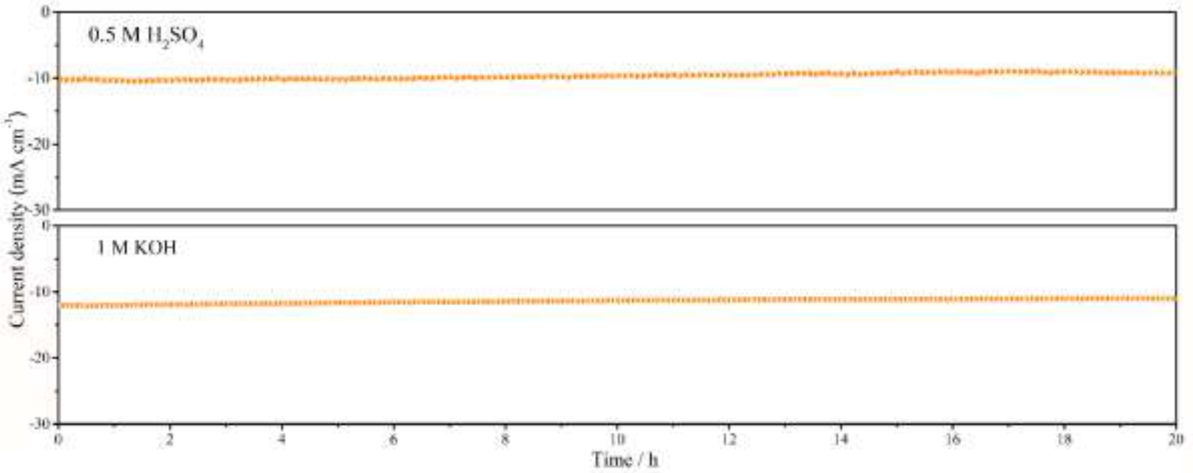

Figure S26. The *i*-t curves of Ru_{SA+NP}/DC in 0.5 M H_2SO_4 and 1 M KOH.

From Figure S26, negligible current losses are observed after continuous HER process over 20 h, demonstrating the excellent long-term durability of Ru_{SA+NP}/DC.

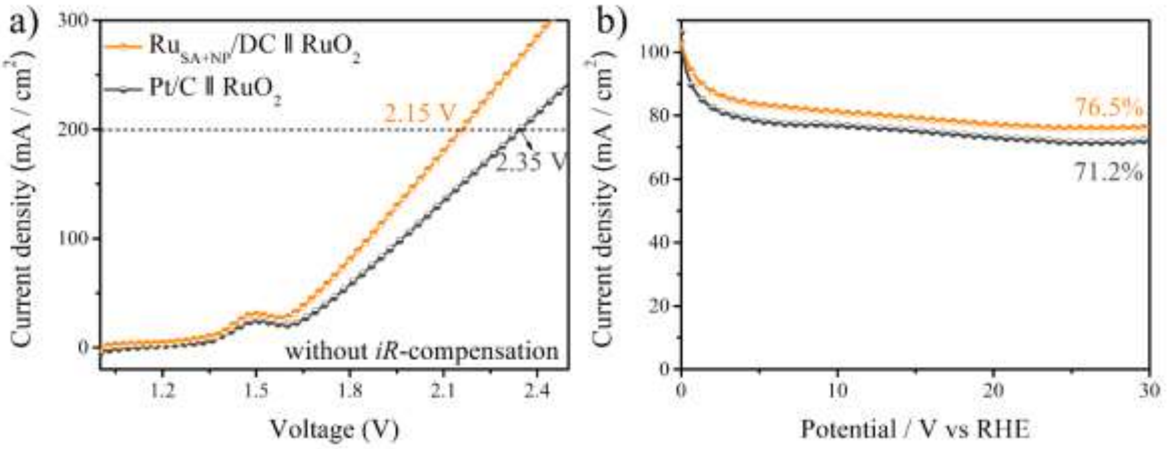

Figure S27. (a) Polarization curves of Ru_{SA+NP}/DC ‖ RuO_2, Commercial Pt/C ‖ RuO_2 coupled catalysts in a two-electrode configuration for overall water splitting in 1.0 M KOH without *iR*-compensation (both loaded into Ni foam at a loading of 2 mg cm^{-2}). (b) Time-dependent current density (i-t) curves for Ru_{SA+NP}/DC ‖ RuO_2, Commercial Pt/C ‖ RuO_2 coupled catalysts in a two-electrode configuration at a certain potential.

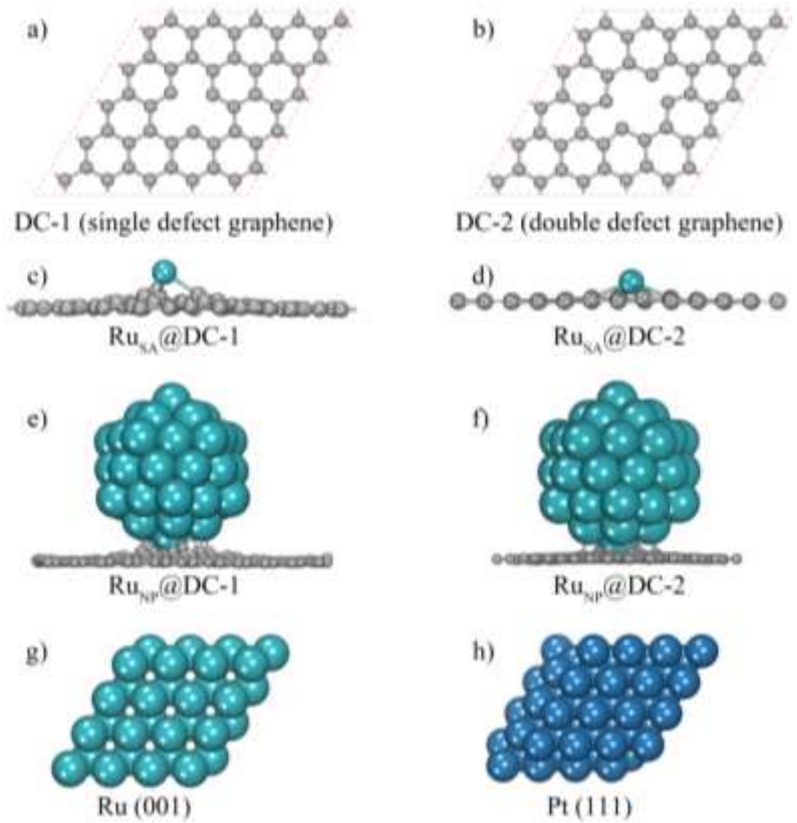

Figure S28. Optimized structural representations of (a) DC-1, (b) DC-2, (c) Ru$_{SA}$@DC-1, (d) Ru$_{SA}$@DC-2, (e) Ru$_{NP}$@DC-1, (f) Ru$_{NP}$@DC-2, (g) Ru (001) and (g) Pt (111)

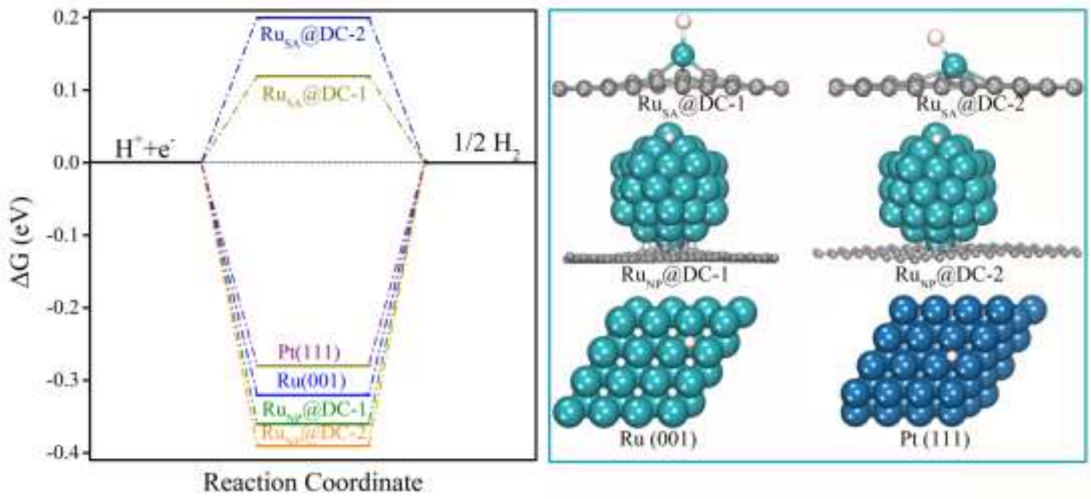

Figure S29. (a) Calculated ΔG_{H^*} for different models. (b) Structures representations of H* adsorbed on different models.

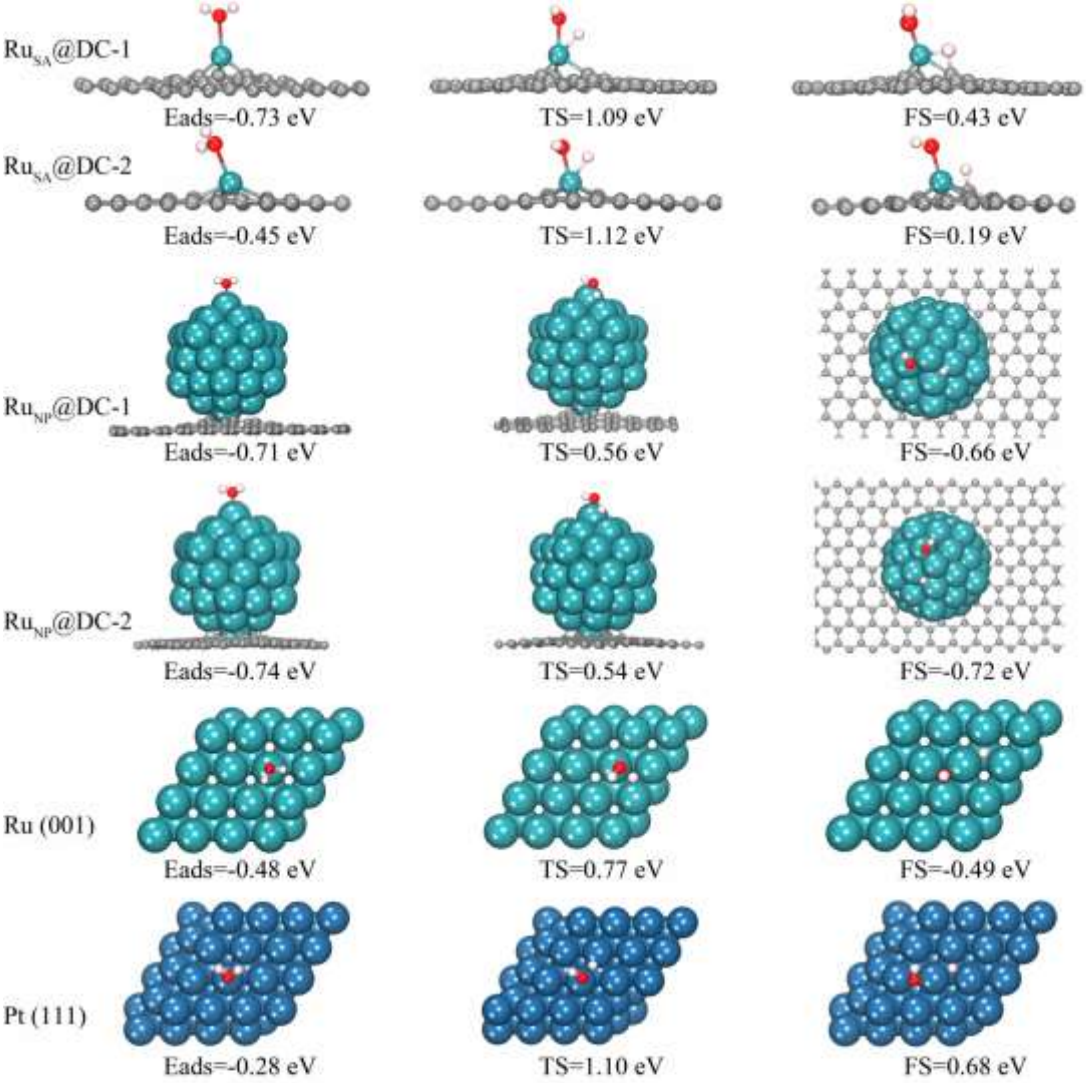

Figure S30. Schematic energy profiles of the dissociation of H_2O by (a) Ru$_{SA}$@DC-1, (b) Ru$_{SA}$@DC-2, (c) Ru$_{NP}$@DC-1, (d) Ru$_{NP}$@DC-2, (e) Ru (001) and (f) Pt (111)

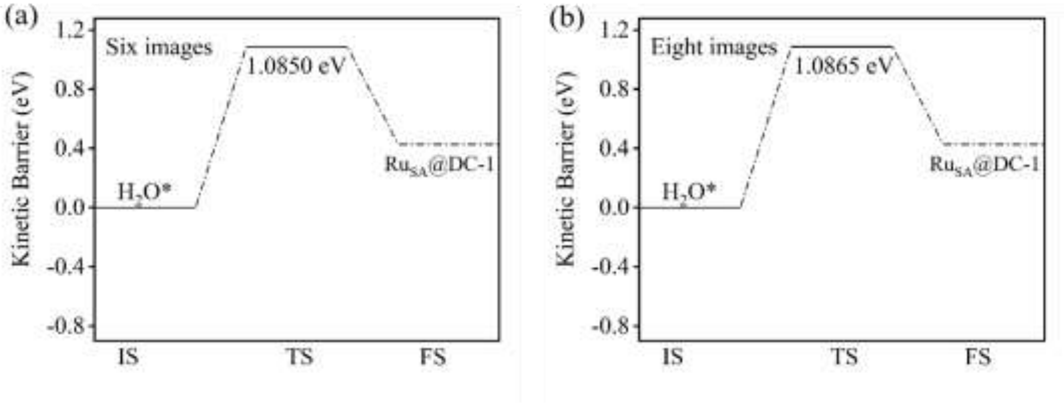

Figure S31. Schematic energy profiles of the dissociation of H_2O by Ru_{SA}@DC-1 performed using the CINEB with 6 images (a) and 8 images (b), respectively.

The H_2O dissociation on Ru_{SA}@DC was calculated using CINEB with 6 and 8 images, respectively. Apparently, a very tiny energy difference of 0.0015 eV is observed, which is negligible for the energy barrier for H_2O dissociation. Therefore, six images are enough for such systems. In order to get results in a reasonable time frame, six images are utilized in this work.

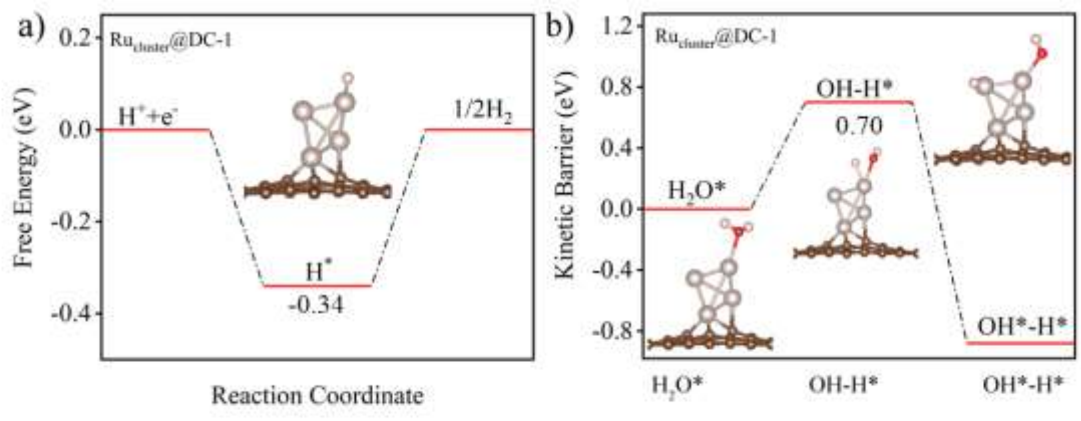

Figure S32. (a) Calculated ΔG_{H*} for Ru$_{cluster}$@DC-1. (b) Kinetic barrier of H2O dissociation for Ru$_{cluster}$@DC-1.

The ΔG_{H*} of Ru$_{cluster}$@DC-1 is −0.34 eV, which is much inferior to that of Ru$_{SA}$@DC-1 (0.12 eV). Compared with that of Ru SA, Ru cluster binds H* too strongly, which may hinder the hydrogen desorption and blocks the active sites, thus leading to the inferior HER activity of Ru cluster to Ru SA in acidic media. The more negative ΔG_{H*} value of Ru$_{cluster}$@DC than that of Ru$_{NP}$@DC-1 (−0.36 eV) suggest that the acidic HER activity of Ru cluster is superior to that of Ru NP. The energy barrier for H_2O dissociation of Ru$_{cluster}$@DC-1 is 0.70 eV, which is much lower than that of Ru$_{SA}$@DC-1 (1.09 eV). Compared with Ru SA, Ru cluster is easier to dissociate H_2O, resulting in the better HER activity of Ru cluster than Ru SA in alkaline media. The H_2O dissociation barrier of Ru$_{cluster}$@DC-1 is higher than that of Ru$_{NP}$@DC-1 (0.56 eV), indicating the inferior HER activity of Ru cluster to Ru NP in alkaline media.

Table S1. The contents of total Ru, Ru NPs, Ru SAs and corresponding decrement in initial Ru_{SA+NP}/DC and samples before and after test in 0.5 M H_2SO_4 and 1 M KOH, respectively.

Sample	Total Ru content (wt.%)	Ru NPs content (wt.%)	Decrement of Ru NPs	Ru SAs content (wt.%)	Decrement of Ru SAs
**Initial**	11.8	8.1	–	3.7	–
**After test in 0.5 M H_2SO_4**	6.3	4.0	50.6%	2.3	37.8%
**After test in 1 M KOH**	6.9	4.8	40.7%	2.1	43.2%

Compared with the initial sample, the Ru NPs and SAs contents in sample after reaction in 0.5 M H_2SO_4 are decreased by 50.6 and 37.8%, respectively. Hence, during the catalytic process in 0.5 M H_2SO_4, the loosing of Ru NPs is more serious than that of Ru SAs, leading to the increase of relative content of Ru SAs. As for the sample after test in 1 M KOH, the Ru NPs and SAs contents in sample after reaction in 1 M KOH are decreased by 40.7 and 43.2%, respectively. Hence, compared with that in 0.5 M H_2SO_4, during the catalytic process in 1 M KOH, the leaching of Ru NPs is alleviated, whereas more Ru SAs are lost, giving rise to the increase of relative content of metallic Ru.

Table S2. The fitting results and parameters for the EXAFS curves of Ru_{SA+NP}/DC catalyst before and after HER test in H_2SO_4 and KOH.

	**Bond**	**Coordination number (CN)**	**Bond length R (Å)**	**σ^2 (Å) x 10^{-3}**	**E_0 (eV)**	**R factor**
**Ru foil**	Ru-Ru	12	2.672	4.0	3.5	0.00184
**Initial sample**	Ru-Ru	10.7	2.67	5.4	5.5	0.00248
	Ru-C	3.2	1.87	5.5	3.5	0.00187
**After test in H_2SO_4**	Ru-Ru	10.9	2.67	4.9	5.4	0.00126
	Ru-C	3.4	1.87	2.4	4.8	0.00252
**After test in KOH**	Ru-Ru	11.6	2.67	5.0	5.4	0.00225
	Ru-C	3.5	1.87	2.3	4.5	0.00228

Table S3. Summary of recently reported HER catalysts in acidic electrolyte.

catalyst	η_{10} (mV)	Reference
Commercial Pt/C	16.5	This work
Ru_{SA+NP}/DC	14.1	This work
Ru@RuN_x NPs	10	Adv. Energy. Mater. 2019, 9, 1900931.
Ru@MWCNT	13	Nat. Commun. 2020, 11, 1278.
Ru@C_2N	13.5	Nat. Nanotech. 2017, 12, 441.
Co-RuIr	14	Adv. Mater. 2019, 31, 1900510.
Pt–GT-1	18	Nat. Energy 2018, 3, 773.
RuB_2	18	Adv. Energy. Mater. 2019, 9, 1803369.
Pt_xNi@Ti_3C_2	18.55	Small 2019, 15, 1805474.
RuCu NSs/C-250 8C	19	Angew. Chem. Int. Ed. 2019, 58, 13983.
Ru_1Ni_1-NCNFs	23	Adv. Sci. 2020, 7, 1901833.
Ru SAs@PN	24	Angew. Chem. Int. Ed. 2018, 130, 9639.
a-$RuTe_2$ PNRs	27	Nat. Commun. 2019, 10, 5692.
hcp-Ru@NC	27	ACS Catal. 2018, 8, 5714.
Ru-NC-700	29	Nat. Commun. 2019, 10, 631.
Ru/C-H_2O/CH_3CH_2OH	35	Appl. Catal. B: Environ. 2019, 258, 117952.
Pt_1/OLC	38	Nat. Energy 2019, 4, 512.
Ru–$Ru_2P\Phi$NPC	42	Adv, Funct. Mater. 2019, 29, 1901154.
RuSi	49	Angew. Chem. Int. Ed. 2019, 58, 11409.
SA Co-D 1T MoS_2	51	Nat. Commun. 2019, 10, 5231.
Ru-MoO_2	54	J. Mater. Chem. A 2017, 5, 5474.
Pt@NHPCP	56	Nano Energy 2017, 40, 88.
Ni-W_2C	57	Research, 2019, 4029516.
RuM/CQDs	58	Angew. Chem. Int. Ed. 2020, 20, 1718.
Amorphous Pd NPs-Bis-24h	60	Adv. Mater. 2020, 32, 1902964.
Ru-MoS_2/CC	61	Appl. Catal. B: Environ. 2019, 249, 91.
PANI/CoP HNWs	65	J. Am. Chem. Soc. 2018, 140, 5118.
PdCu@Pd nanocubes	68	ACS Appl. Mater. Interfaces 2017, 9 8151.
aNi@G585	70	Chem 2018, 4, 285.
Pt@MTO-S	73	ChemCatChem 2019, 11, 583.
RuSA-N-S-Ti_3C_2Tx	76	Adv. Mater. 2019, 31, 1903841.
Au@Pd1.0 nanoparticles	79	Int. J. Electrochem. Sci 2020, 15, 2634.

PdCu nanocrystals	80	ACS Appl. Mater. Interfaces 2019, 11, 3861.
$(Fe_{0.048}Ni_{0.952})_2P$ nanosheets	81	Nano Energy 2019, 56, 813.
W-CoP NAs/CC	89	Small 2019, 15, 1902613.
Li-$Pd_3P_2S_8$	91	Nat. Catal. 2018, 1, 460.
1D-RuO_2-CN_x	93	ACS Appl. Mater. Interfaces 2016, 8, 28678.
MoP@NC	96	Appl. Catal. B: Environ. 2019, 245, 656.
Ru/MoS_2/CP	97	Nanoscale 2017, 9, 16616.
Co@Pd NC	98	ACS Catal. 2015, 5, 5264.
MoS_2 NAs/Ti	108	Electrochim. Acta 2015, 168, 256.
C/N-co-doped Ag@Pd NWs	111	Electrochim. Acta 2018, 283, 221.
0.02Ni–MoP	112	Nano Energy 2020, 70, 104445.
CoP_3/Ni_2P	115	J. Mater. Chem. A, 2018, 6, 5560.
$Cu_{2-x}S$@Ru NPs	131	Small 2017, 13, 1700052.
CoN_x/C	130	Nat. Commun. 2015, 6, 7992.
Mo_2C@C	140	Small 2017, 13, 1701246.
MoP/NPG	148	ChemElectroChem 2018, 5, 2256.
MoS_2 Nanoscroll	153	ACS Energy Lett. 2019, 4, 12, 2830.
Edge-terminated MoS2	153	Nat. Commun 2015, 6, 7493.
$MoSe_2$-Co_2P	167	Reasearch, 2019, 6439734.
Au@MoS_2	178	Nano Res. 2019, 12, 1301.
Ag@MoS_2	195.7	J. Am. Chem. Soc. 2020, 142, 7161.
C_3N_4@NG	240	Nat. Commun. 2014, 5, 3783.

Table S4. Summary of recently reported HER catalysts in alkaline electrolyte.

catalyst	η_{10} (mV)	Reference
Commercial Pt/C	32.2	This work
Ru_{SA+NP}/DC	15	This work
Ru@RuN_x NPs	7	Adv. Energy. Mater. 2019, 9, 1900931.
Rh NP/C	7	Adv. Energy. Mater. 2018, 5, 1870135.
Ru@CQDs	10	Adv. Mater. 2018, 30, 1800676.
Ru-NC-700	12	Nat. Commun. 2019, 10, 631.
RuM/CQDs	13	Angew. Chem. Int. Ed. 2020, 20, 1718.
Co substituted Ru NSs	13	Nat. Commun. 2018, 9, 4958.
Ru/C-300	14	J. Mater. Chem. A 2018, 6, 14380.
RuNi NS	15	Nano Energy 2019, 66, 104173.
Ru@MWCNT	17	Nat. Commun. 2020, 11, 1278.
Ru@C2N	17	Nat. Nanotech. 2017, 12, 441.
L-RuP/C	18	Adv. Mater. 2018, 30, 1800047.
RuCu NSs/C-250 8C	20	Angew. Chem. Int. Ed. 2019, 58, 13983.
Ru@GnP	22	Adv. Mater. 2018, 30, 1803676.
4H/fcc Ru NTs	23	Small 2018, 14, 1801090.
RuAu-0.2	24	Adv. Energy. Mater. 2019, 9, 1803913.
Ru@NC	27	Angew. Chem. Int. Ed. 2018, 130, 5950.
Cu-doped Ru-RuO_2/C	27	Small 2018, 14, 1803009.
RuCo@NC	28	Nat. Commun. 2017, 8, 14969.
RuB_2	28	Adv. Energy. Mater. 2019, 9, 1803369.
NiFeRu-LDH	30	Adv. Mater. 2018, 30, 1706279.
Ru@CN-0.16	32	Energy Environ. Sci., 2018, 11, 800.
CoP/CoMoP	34	Nano Energy 2020, 68, 104332.
Ru-MoO_2	35	J． Mater. Chem. A 2017, 5, 5474.
Ru1Ni1-NCNFs	35	Adv. Sci. 2020, 7, 1901833.
a-$RuTe_2$ PNRs	41	Nat. Commun. 2019, 10, 5692.
Ru-MoS_2/CC	41	Appl. Catal. B: Environ. 2019, 249, 91.
Pd_3Ru/C	42	ACS Catal.2019, 9, 9614.
CoP–CeO_2/Ti	43	J. Mater. Chem. A, 2018, 6, 1985.
Ni–Mo–N/NG	46.6	Carbon 2020, 165, 122.

Ru_2P@PNC/CC-900	50.7	ACS Appl. Energy Mater. 2018, 1, 3143.
Ru/C-H_2O/CH_3CH_2OH	53	Appl. Catal. B: Environ. 2019, 258, 117952.
Ni_5P_4-Ru	54	Adv. Mater. 2020, 32, 1906972.
Ir_1@Co/NC	55	Angew. Chem. Int. Ed. 2019, 131, 11994.
CS-MoS_2	56	Appl. Surface Sci. 2019, 463, 182.
Sr_2RuO_4	61	Nat. Commun. 2019, 10, 149.
np-Ni_3N	68	Angew. Chem. Int. Ed. 2020, 59, 10797.
Ni-CMB	69	Nano Energy 2020, 67, 104245.
V-Ce/CoFe LDH	73	J. Mater. Chem. A, 2020, 8, 2490.
CoP/CNTs	77	Adv. Funct. Mater. 2017, 27, 1606635.
Co-NiS_2 NSs	80	Angew. Chem. Int. Ed. 2019, 131, 18849.
$Cu_{2-x}S$@Ru NPs	82	Small 2017, 13, 1700052.
W-CoP NAs/CC	94	Small 2019, 15, 1902613.
$(Fe_{0.048}Ni_{0.952})_2P$ nanosheets	103	Nano Energy 2019, 56, 813.
Ni–Co–P	107	Energy Environ. Sci., 2018, 11, 872.
$Ni_{1.5}Co_{0.5}$@N-C NT/NF	114	Adv. Sci. 2020, 7, 1902371.
$Ni_{11}(HPO_3)_8(OH)_6$/NF	121	Energy Environ. Sci., 2018, 11, 1287.
MoP/NPG	126	ChemElectroChem 2018, 5, 2256.
R-NCO	135	J. Am. Chem. Soc. 2018, 140, 13644.
MoC_x	149	Nat. Commm. 2015, 6, 6512.
TiO_2:Ru (5%)	150	J. Am. Chem. Soc. 2018, 140, 5719.
$Fe_2P_2S_6$ NCs	175	Small Methods 2020, 4, 1900632.
Co-Ni_3N	194	Adv. Mater. 2018, 30, 1705516.

Table S5. A survey of Ru SAs and NPs-based HER electrocatalysts with several parameters of elements and structures, water dissociation barrier and η_{10} values in acidic and alkaline media.

catalyst	Elements and structure	Water dissociation barrier	η_{10} (mV) acidic	alkaline	refs
Ru_{SA+NP}/DC	Ru, C and O;<br>Ru SAs and NPs on defective carbon	0.54 eV for Ru NP@ defective graphene	14.1	16.5	This work
Ru-NC-700	Ru, C, N and O;<br>Ru SAs and NPs on N-doped carbon nanowires	0.88 eV for RuC_2N_2	29	12	12
[Ru(SA)+ Ru(NP)@RuN*x*@GN]/GN	Ru, C, N and O;<br>Ru SAs and N-doped graphitic-shell covered nitride-Ru NPs (having a $Ru-N_x$ shell)	None	12	8	13
Ru@CQDs	Ru, C, N and O;<br>Ru NPs supported on carbon quantum dots	1.94 eV for Ru@graphene	None	10	14
Ru@GnP	Ru, C and O;<br>Ru NPs anchored on graphene nanoplatelets,	None	13	22	15
Ru/C-H_2O/CH_3CH_2OH	Ru, C and O;<br>Ru NPs on carbon matrix	None	35	53	16
Ru/NC	Ru, C and O;<br>Ru SAs and NPs on N-doped carbon	None	None	21	17
Ru SA@PN	Ru, P, N and C;<br>Ru SAs supported on HPN	None	24	~ 80	18
Ru@C_2N	Ru, C, N and O;<br>Ru NPs within a nitrogenated holey 2D carbon structure	None	13.5	17	19
Ru@CN	Ru, C, N and O;<br>Ru NPs on N-doped carbon	None	None	32	20
hcp-Ru@ NC	Ru, C, N and O;<br>Ru NPs on N-doped carbon	None	27	None	21
TiO_2:Ru (5%)	Ru, Ti and O;<br>Ru is doped into the lattice of TiO_2 forming RuO_2-TiO_2 solid solution	None	None	150	22
RuSA-N-S -Ti_3C_2Tx	Ru, C, N, S,and Ti;<br>Ru SA coordinated with N and S sites on $Ti_3C_2T_x$	None	76	~ 190	23
RuAu SSA	Au and Ru;<br>Isolated Au atoms in Ru NPs	None	None	24	24
ECM@Ru	Ru, C, N and O;<br>Isolated Ru atom on edge-rich carbon matrix	None	63	83	25
SA-Ru -MoS_2	Ru, Mo and S;<br>Ru single atoms embedded in 2D MoS_2 plane	-0.09 eV for 1T-Ru-MoS_2 -S_v	None	76	26

References

[1] Y. Zou, S. Chen, X. Yang, N. Ma, Y. Xia, D. Yang, S. Guo, *Adv. Energy Mater.* **2016**, *6*, 1601549.

[2] D. Li, C. Lv, L. Liu, Y. Xia, X. She, S. Guo, D. Yang, *ACS Cent. Sci.* **2015**, *1*, 261.

[3] K. Wu, L. Zhang, Y. Yuan, L. Zhong, Z. Chen, X. Chi, H. Lu, Z. Chen, R. Zou, T. Li, C. Jiang, Y. Chen, X. Peng, J. Lu, *Adv. Mater.* **2020**, *32*, 2002292.

[4] S. Wei, A. Li, J. C. Liu, Z. Li, W. Chen, Y. Gong, Q. Zhang, W. C. Cheong, Y. Wang, L. Zheng, H. Xiao, C. Chen, D. Wang, Q. Peng, L. Gu, X. Han, J. Li, Y. Li, *Nature Nanotech.* **2018**, *13*, 856.

[5] Y. Zhang, L. Gao, E. J. M. Hensen, J. P. Hofmann, *ACS Energy Lett* **2018**, *3*, 1360.

[6] L. Zhang, Y. Jia, G. Gao, X. Yan, N. Chen, J. Chen, M. T. Soo, B. Wood, D. Yang, A. Du, X. Yao, *Chem* **2018**, *4*, 194.

[7] G. Kresse, J. Furthmüller, *Phys. Rev. B* **1996**, *54*, 11169

[8] J. P. Perdew, K. Burke, M. Ernzerhof, *Phys. Rev. Lett.* **1996**, *77*, 3865.

[9] G. Henkelman, H. Jónsson, *J. Chem. Phys.* **2000**, *113*, 9978.

[10] G. Henkelman, B. P. Uberuaga, H. Jónsson, *J. Chem. Phys.* **2000**, *113*, 9901.

[11] J. K. Nørskov, J. Rossmeisl, A. Logadottir, L. Lindqvist, J.R. Kitchin, T. Bligaard, H. Jonsson, *J. Phys. Chem. B* **2004**, *108*, 17886.

[12] B. Lu, L. Guo, F. Wu, Y. Peng, J. E. Lu, T. J. Smart, N. Wang, Y. Z. Finfrock, D. Morris, P. Zhang, N. Li, P. Gao, Y. Ping, S. Chen, *Nat.Commun.* **2019**, *10*, 631.

[13] J. N. Tiwari, A. M. Harzandi, M. Ha, S. Sultan, C. W. Myung, H. J. Park, D. Y. Kim, P. Thangavel, A. N. Singh, P. Sharma, S. S. Chandrasekaran, F. Salehnia, J. W. Jang, H. S. Shin, Z. Lee, K. S. Kim, *Adv. Energy Mater.* **2019**, *9*, 1900931.

[14] W. Li, Y. Liu, M. Wu, X. Feng, S. A. T. Redfern, Y. Shang, X. Yong, T. Feng, K. Wu, Z. Liu, B. Li, Z. Chen, J. S. Tse, S. Lu, B. Yang, *Adv. Mater.* **2018**, *30*, 1800676.

[15] F. Li, G. F. Han, H. J. Noh, I. Ahmad, I. Y. Jeon, J. B. Baek, *Adv. Mater.* **2018**, *30*, 1803676.

[16] Y. Li, J. Abbott, Y. Sun, J. Sun, Y. Du, X. Han, G. Wu, P. Xu, *Appl. Catal. B: Environ.* **2019**, *258*, 117952.

[17] J. Zhang, P. Liu, G. Wang, P. P. Zhang, X. D. Zhuang, M. W. Chen, I. M. Weidinger, X. L. Feng, *J. Mater. Chem. A* **2017**, *5*, 25314.

[18] J. Yang, B. Chen, X. Liu, W. Liu, Z. Li, J. Dong, W. Chen, W. Yan, T. Yao, X. Duan, Y. Wu, Y. Li, *Angew. Chem. Int. Ed.* **2018**, *57*, 9495.

[19] J. Mahmood, F. Li, S. M. Jung, M. S. Okyay, I. Ahmad, S. J. Kim, N. Park, H. Y. Jeong, J. B. Baek, *Nat. Nanotech.* **2017**, *12*, 441.

[20] J. Wang, Z. Wei, S. Mao, H. Li, Y. Wang, *Energy Environ. Sci.* **2018**, *11*, 800.

[21] Y. Li, L. A. Zhang, Y. Qin, F. Chu, Y. Kong, Y. Tao, Y. Li, Y. Bu, D. Ding, M. Liu, *ACS Catal.* **2018**, *8*, 5714.

[22] S. Nong, W. Dong, J. Yin, B. Dong, Y. Lu, X. Yuan, X. Wang, K. Bu, M. Chen, S. Jiang, L. M. Liu, M. Sui, F. Huang, *J. Am. Chem. Soc.* **2018**, *140*, 5719.

[23] V. Ramalingam, P. Varadhan, H. C. Fu, H. Kim, D. Zhang, S. Chen, L. Song, D. Ma, Y. Wang, H. N. Alshareef, J. H. He, *Adv. Mater.* **2019**, *31*, 1903841.

[24] C. H. Chen, D. Wu, Z. Li, R. Zhang, C. G. Kuai, X. R. Zhao, C. K. Dong, S. Z. Qiao, H. Liu, X. W. Du, *Adv. Energy Mater.* **2019**, *9*, 1803913.

[25] H. Zhang, W. Zhou, X. F. Lu, T. Chen, X. W. Lou, *Adv. Energy Mater.* **2020**, *10*, 2000882.

[26] J. Zhang, X. Xu, L. Yang, D. Cheng, D. Cao, *Small Methods* **2019**, *3*, 1900653.
